# Health economic evaluations for Indonesia: a systematic review assessing evidence quality and adherence to the Indonesian Health Technology Assessment (HTA) Guideline

**DOI:** 10.1016/j.lansea.2023.100184

**Published:** 2023-03-31

**Authors:** Kinanti Khansa Chavarina, Dian Faradiba, Ella Nanda Sari, Yi Wang, Yot Teerawattananon

**Affiliations:** a Saw Swee Hock School of Public Health, National University of Singapore, Singapore; b Health Intervention and Technology Assessment Program (HITAP), Ministry of Public Health, Bangkok, Thailand

**Keywords:** Indonesia, Health economic evaluation, Appraisal, National guideline

## Abstract

**Background:**

The Government of Indonesia implemented health technology assessment (HTA) to ensure quality and cost control in the National Health Insurance Program (*Jaminan Kesehatan Nasional*/JKN). The current aim of the study was to improve the usefulness of future economic evaluation for resource allocation by appraising current methodology, reporting, and source of evidence quality of studies.

**Methods:**

The inclusion and exclusion criteria were applied to search for relevant studies using a systematic review. The methodology and reporting adherence were appraised according to Indonesia's HTA Guideline issued in 2017. The differences in adherence before and after the guideline dissemination were compared using Chi-square and Fisher's exact tests for methodology adherence wherever appropriate, and the Mann–Whitney test for reporting adherence. The source of evidence quality was assessed using evidence hierarchy. Two scenarios of the study start date and the guideline dissemination period were tested using sensitivity analyses.

**Findings:**

Eighty-four studies were obtained from PubMed, Embase, Ovid, and two local journals. Only two articles cited the guideline. No statistically significant difference (P > 0.05) was found between the pre-dissemination and post-dissemination period with respect to methodology adherence, except for outcome choice. Studies during the post-dissemination period showed a higher score for reporting which was statistically significant (P = 0.01). However, the sensitivity analyses revealed no statistically significant difference (P > 0.05) in methodology (except for modelling type, P = 0.03) and reporting adherence between the two periods.

**Interpretation:**

The guideline did not impact the methodology and reporting standard used in the included studies. Recommendations were provided to improve the usefulness of economic evaluations for Indonesia.

**Funding:**

The Access and Delivery Partnership (ADP) hosted by the 10.13039/100016195United Nations Development Programme (UNDP) and the 10.13039/501100010724Health Systems Research Institute (HSRI).

**Research in context**

**Evidence before this study**

There has been increasing use of health technology assessment (HTA), especially in informing coverage decisions of medical technology in low-income and middle-income countries. As such, local governments often issue HTA guidelines to ensure the quality, comparability, and transparency of commissioned studies. A recent systematic review demonstrates that adherence to country-specific guidelines can enhance the quality of local economic evaluations. As such, there are requests to funders and the research community for greater adherence to the methodological guidelines. Nevertheless, the previous reviews show varying results on adherence of HTA studies to their relevant guidelines.

**Added value of this study**

Moving from HTA guideline development to implementation is a vital step toward improving the quality of evidence-informed policy. This is the first study that appraised health economics evaluation for Indonesia. This study shows how the Indonesia HTA guideline has made an impact on improvement in methodology and reporting standards of local studies after its dissemination.

**Implications of all the available evidence**

This study attempts to support Indonesia's HTA development by providing recommendations to the government, researchers, and funders on improving the use of health economic evaluation for reimbursement decision-making. Future efforts could include additional educational activities for researchers and policymakers. Policymakers, HTA funders, and journals could strictly request that researchers adhere to relevant guidelines. Lastly, we also encourage using a similar approach and framework for evaluating adherence to HTA guidelines in other settings.

## Introduction

In 2012, The Roadmap of the *Jaminan Kesehatan Nasional* (JKN), or National Health Insurance program for 2012–2019 was issued as a commitment from the Indonesian government to provide national health insurance.^1^ It depicts Indonesia's ambitious, single-payer program launched in 2014, and implemented by a legal entity, *Badan Penyelenggara Jaminan Kesehatan* (BPJS).^1^^,^^2^ The government's strong commitment was paid off as it covered 241.7 million people of Indonesia (as of June 2022). All enrolees were guaranteed generous benefit packages, for example, health promotion and diagnostic tests as well as preventive, curative, rehabilitative, and ambulance services.^3^ The list of drugs in the benefit packages is detailed in the national formulary issued and updated regularly by the Ministry of Health, Indonesia.^4^

To support the JKN sustainability while delivering qualified benefit packages, the government stipulated the implementation policy of the health technology assessment (HTA) in Presidential Decree No. 82, 2018.^5^ Prior to this decree, the Ministry of Health formed an HTA Committee (InaHTAC) in 2014 as the centre of HTA activity in Indonesia, including developing policy recommendations to the Ministry of Health to validate the BPJS decision.^6^^,^^7^ The HTA activities in Indonesia gradually gained momentum since the establishment of the InaHTAC, such as biennial scientific meetings and collaborations with local and international partners for studies, knowledge sharing, and policy forums.^8^ However, there are remaining challenges that hinder the development of HTA, such as inadequate financial incentives, data infrastructure, local capacity and capability, and a gap in the evidence translation into the policy-making process.^9^

One of the Ministry of Health's efforts to implement HTA was a pharmacoeconomic guideline issued in December 2012.^10^ It provides general theory, assessment, and appraisal process of pharmacoeconomic studies.^10^ In April 2017, the Indonesian HTA Guideline (mentioned as ‘the guideline’ from now on), was issued by InaHTAC with a more comprehensive theory and methodology reference cases to ensure methodological rigor and reporting quality.^11^ The guideline serves as a resource and material for all stakeholders involved in the JKN in conducting economic evaluations according to the Indonesian context.^11^ It adapts the 2013 Consolidated Health Economic Evaluation Reporting Standards (CHEERS), a checklist that is commonly applied for health economic evaluation reporting.^12^ Despite the recommendations on the methodology and reporting standards, the guideline does not explicitly mention whether economic evaluations for Indonesia must comply with its references to be considered for the reimbursement decision-making process, in contrast to some other countries national health economic evaluation guidelines.^13^ The Ministry of Health Decree No. 51, 2017 which regulates the health technology assessment process also did not mention the use of the reference cases in the guideline or pharmacoeconomic guideline.^14^

The magnitude and scale of JKN as well as limited financial resources that were further undermined by the COVID-19 pandemic urge the government to manage its expenditure wisely.^3^^,^^15^ Appropriate assessment and transparency of health economic evaluation are imperative to ensure efficient funding while driving optimal health outcomes for JKN participants. Therefore, the guideline plays a crucial role as a standard to guide researchers in assessing relevant health technologies according to the Indonesian context. Otherwise, studies may be using irrelevant methodology and reporting standards which decrease their usefulness in the decision-making process of resource allocation.^13^^,^^16^ This is a globally relevant issue given that more countries have issued national HTA guidelines to support HTA implementation in informing universal health coverage policy.^17^

The current study reviewed the methodology, source of evidence quality, and reporting adherence of the health economic evaluations for Indonesia to the guideline's recommendations. The guideline was chosen as a reference because it is a more recent document produced by the national HTA agency and provides more comprehensive reference cases compared to the pharmacoeconomic guideline by the Ministry of Health, Indonesia. To date, there are no published articles on the improvement in methodological and reporting relevance of health economic evaluations to the Indonesian context after the guideline was disseminated.

## Methods

The protocol for this study is registered to PROSPERO under registration ID CRD42021292268. The Preferred Reporting Items for Systematic reviews and Meta-Analyses (PRISMA) 2020 statement was used as a reference for reporting this study.^18^

### Search strategy

Multiple search strategies were tested and refined to identify relevant studies (see Supplementary Material). The final search strategy was chosen by three researchers (KKC, DF, and YT) and PubMed, Embase, and Ovid databases searched in December 2021. The following keywords in the search term were matched to the indexing or classification of economic evaluation between databases: ((Cost) OR (Cost Analysis) OR (Cost Savings) OR (Cost of Illness) OR (Cost Benefit) OR (Cost-benefit) OR (Cost Utility) OR (Cost-utility) OR (Cost Effectiveness) OR (Cost-effectiveness) OR (Cost Minimisation) OR (Cost-minimisation) OR (Cost Minimization) OR (Cost-minimization) OR (Economic Analysis) OR (Economic Evaluation)) AND (Indonesia).

There was no comprehensive database for local health journals; therefore, no information was retrieved on the name of journals that published health economic evaluations. The search for local journals resorted to an active local community, the Indonesian Health Economics Association, which regularly conducts congress on health economics. Abstracts and posters submitted to the congress have been published in two local journals, *Jurnal Manajemen Kesehatan Indonesia* (*JMKI*) and *Jurnal Ekonomi Kesehatan Indonesia* (*JEKI*). These two journals were chosen to search for locally published studies. Each volume was screened manually for title and abstract as the journal databases were unable to handle search terms.

### Study eligibility and systematic review process

We selected peer-reviewed articles that evaluated health technologies for Indonesia in English or Indonesian language. This included multi-country studies with Indonesia as part of it. The guideline only provides recommendations for full health economic evaluation; therefore, only full health economic evaluations were included. Articles were considered full health economic evaluations if they evaluated the costs and consequences of the health interventions and compared to at least two alternatives.^19^ These alternatives can include a no-intervention scenario in the analysis.^19^ All studies up to December 2021 (search date) with any population, intervention, and comparator were included.

Studies for which full-text was unavailable, posters, editorial, methodological, commentaries, case studies, brief communications, and review articles were excluded. Systematic reviews of health economic evaluations were also excluded because it has a different methodology compared to regular economic evaluation and the guideline does not provide methodology recommendations on conducting such study design.

The reference management software Covidence was used and two researchers (KKC and DF) screened identified titles and abstracts independently. Conflicts were handled through discussions. The third researcher (YT) conducted the arbitration in the case of no consensus. Full-text articles of potential studies were reviewed in detail regarding eligibility criteria by two researchers (KKC and DF). The remaining studies were extracted by three researchers (KKC, DF, and ENS).

### Data extraction

An extraction form was developed containing general information (first author name, first author's affiliation, publication type, journal name, publication year, funders, language, disease type, technology type, study objective), methodology specifications, reporting parameters, and source of evidence used.

Methodology recommendations were extracted from the guideline and categorised into methodology ‘specification’ and ‘guidance’. Briefly, methodology specification refers to specific recommendations that can be directly measured across studies. For example, the guideline recommends a 3% discount rate for costs and outcomes. Methodology adherence (hereinafter, extraction on methodology specification is referred to as methodology adherence) was recorded as ‘yes’, ‘no’, or ‘not applicable.’ Methodology guidance refers to guiding principles recommended to help researchers in adopting their methodological or data choices used in the study. For example, the guideline recommends developing a theoretical and conceptual framework using systematic review results. The extractions and analysis for methodological guidance were merged with either reporting adherence or source of evidence quality. For detailed information, Table 3 in Supplementary Material provide a full list of methodology specification and guidance used in the data extraction of this review.

Reporting parameters were based on the 2013 CHEERS checklist, as recommended by the guideline.^11^^,^^12^ In the CHEERS items, the measurement of effectiveness, estimate of resources and cost, and uncertainty analysis items are divided into single study-based economic evaluation (or evaluations using clinical study) and model-based economic evaluation. Information on reporting adherence was recorded as ‘yes,’ ‘no,’ or ‘not applicable.’ For a CHEERS item that contains more than one parameter, adherence was recorded as ‘yes’ if studies reported at least one parameter within the item.

The source of evidence quality was ranked based on potential hierarchies of data sources by Cooper and colleagues, a widely used instrument to assess evidence quality in economic evaluations.^20^ Disagreements in extraction results were resolved through discussion and consultation with the fourth researcher (YT).

### Data analysis

Descriptive statistics were summarised in tables and figures, detailing the frequency of characteristics in absolute numbers and proportion. Study adherence before and after the guideline dissemination was compared. The studies were categorised into pre-dissemination period and post-dissemination period based on the start date of the study. The assumption of the start date of the study was one year before publication unless stated otherwise. As the guideline was published in 2017, studies started in 2019–2021 were categorised in the post-dissemination period (post-2018), assuming the dissemination period takes two years. For methodology adherence, Chi-square and Fisher's exact tests were used to evaluate differences in the proportion of study within the pre-2018 and post-2018 following the methodology recommendations, wherever appropriate. For reporting adherence, a composite quality score was generated for each study by counting the number of ‘yes’ out of the applicable parameters to compare studies in pre-and post-2018. The scoring range is 0–1. The Mann–Whitney test was used to evaluate the mean ranks as the distribution of studies in pre-2018 and post-2018 has different variability.^21^

The source of evidence was ranked according to the evidence hierarchy by Cooper and colleagues, modified from Coyle and Lee, as seen in Table 1.^20^^,^^22^ Studies with multiple data sources with different evidence ranks were scored for the highest evidence rank available.

**Table 1:** Source of evidence hierarchy according to Cooper et al., modified from Coyle and Lee.^20^

Rank	Data components
	**Clinical effect sizes, adverse events & complications**
1+	Meta-analysis of RCTs with direct comparison between comparator therapies, measuring final outcomes
1	Single RCT with direct comparison between comparator therapies, measuring final outcomes
2+	Meta-analysis of RCTs with direct comparison between comparator therapies, measuring surrogate outcomes Meta-analysis of placebo-controlled RCTs with similar trial populations, measuring the final outcomes for each individual therapy
2	Single RCT with a direct comparison between comparator therapies, measuring surrogate outcomes Single, placebo-controlled, RCTs with similar trial populations, measuring the final outcomes for each individual therapy
3+	Meta-analysis of placebo-controlled RCTs with similar trial populations, measuring the surrogate outcomes
3	Single, placebo-controlled, RCTs with similar trial populations, measuring the surrogate outcomes for each individual therapy
4	Observational studies (e.g., case control)
5	Non-analytic studies (e.g., case reports)
6	Expert opinion
9	Not stated
	**Baseline clinical data**
1	Case series or analysis of reliable administrative databases specifically conducted for the study covering patients solely from the jurisdiction of interest
2	Recent case series or analysis of reliable administrative databases covering patients solely from the jurisdiction of interest
3	Recent case series or analysis of reliable administrative databases covering patients solely from another jurisdiction
4	Old case series or analysis of reliable administrative databases; estimate from RCTs
5	Estimates from previously published economic analyses: unsourced
6	Expert opinion
9	Not stated
	**Resource use**
1	Prospective data collection or analysis of reliable administrative data for specific study
2	Recently published results of prospective data collection or recent analysis of reliable administrative data—same jurisdiction
3	Unsourced data from previous economic evaluations—same jurisdiction
4	Recently published results of prospective data collection or recent analysis of reliable administrative data—different jurisdiction
5	Unsourced data from previous economic evaluations—different jurisdiction
6	Expert opinion
9	Not stated
	**Costs**
1	Cost calculations based on reliable databases or data sources conducted for specific study—same jurisdiction
2	Recently published cost calculations based on reliable databases or data course—same jurisdiction
3	Unsourced data from previous economic evaluation—same jurisdiction
4	Recently published cost calculations based on reliable databases or data course—different jurisdiction
5	Unsourced data from previous economic evaluation—different jurisdiction
6	Expert opinion
9	Not stated
	**Utilities**
1	Direct utility assessment for the specific study from a sample either: (a) of the general population, (b) with knowledge of the disease(s) of interest, (c) of patients with the disease(s) of interest Indirect utility assessment for the specific study from patient sample with disease(s) of interest, using a tool validated for the patient population
2	Indirect utility assessment for the specific study from patient sample with disease(s) of interest, using a tool not validated for the patient population
3	Direct utility assessment from a previous study from a sample either: (a) of the general population, (b) with knowledge of the disease(s) of interest, (c) of patients with the disease(s) of interest
4	Unsourced utility data from previous study—method of elicitation unknown
5	Patient preference values obtained from a visual analogue scale
6	Delphi panels, expert opinion
9	Not stated

RCT: Randomised controlled trial.

The regression framework was applied to understand which study characteristics independently affect the scores of methodology and reporting.^21^ The study characteristics were 1) first author affiliation, 2) involvement of foreign affiliation, 3) source of funding, 4) disease category, 5) type of technology, and 6) study design (clinical study or model-based evaluation). Study characteristics with a dominant proportion of study in its sub-characteristics were omitted, and some sub-characteristics were regrouped to avoid multicollinearity issues (see Supplementary Material). The scores of methodology and reporting were generated for each study using the same composite quality score method as explained above. Univariate fractional logistic regressions were applied first then followed by multivariable fractional logistic regressions. Due to the relatively small sample size, a forward stepwise selection process was used to identify the final model. Variables were included one by one from the highest log pseudolikelihood to the lowest log pseudolikelihood from the univariate fractional logistic regression. The forward stepwise selection stopped when the newly added variable was not significant at 10%.

Two sensitivity analyses were conducted for methodology and reporting adherence. Different assumptions on the time taken for guideline dissemination and the start date of the study were considered for testing the robustness of results. First, it was assumed that dissemination of the guideline takes only one year. Studies after 2017 (2018–2021) were included in the post-dissemination period, and studies in 2017 and before were included in the pre-dissemination period. Second, it was assumed that dissemination takes no time and the study start date was two years before publication. Studies started in 2017 onwards were included in the post-dissemination period.

### Role of the funding source

The funding agency had no role in study design, data collection, data analysis, interpretation, or writing of the report.

## Results

**Fig. 1:**
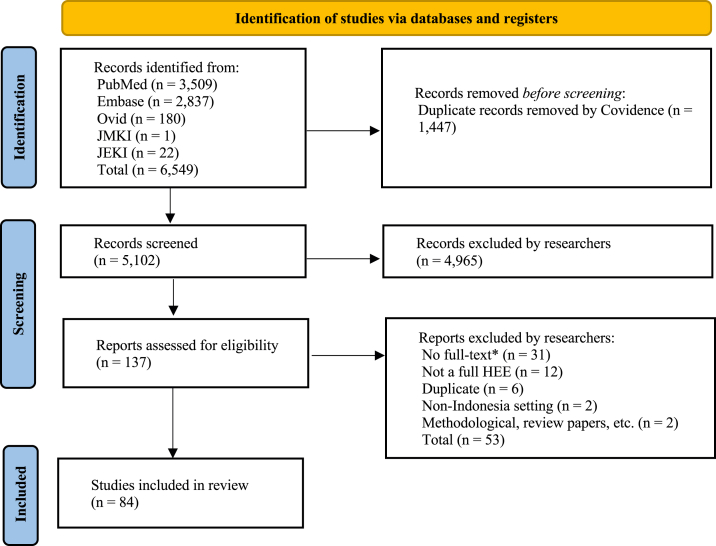
PRISMA flow diagram.^18^

The search from PubMed, Embase, Ovid, JMKI, and JEKI fetched 6549 records. The Covidence software automatically removed the duplicates and 5102 records were screened for the title, abstracts, and full-text review according to the inclusion and exclusion criteria. Majority of the abstracts did not match our inclusion criteria and 4965 abstracts were excluded. Studies related to Indonesia, but which did not evaluate the health technologies for the Indonesian setting were also excluded. For example, a study by Massad and colleagues was excluded as it evaluated the chemo-prophylactic policy for UK travellers traveling to malaria-endemic regions including Indonesia.^23^ Duplicates were further assessed manually at the final stage of the full-text review, which resulted in 84 eligible studies (Fig. 1, see Supplementary Material).

**Fig. 2:**
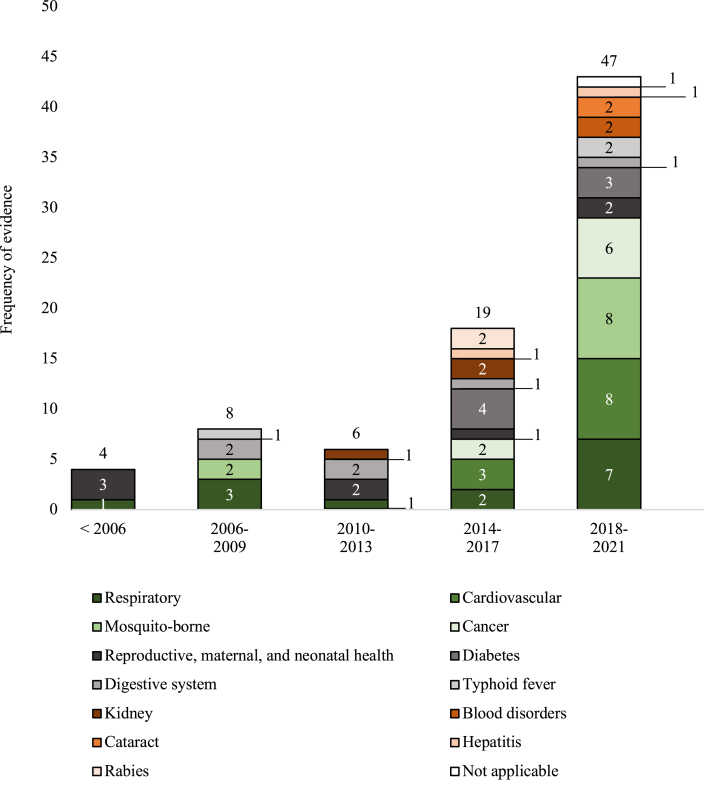
Frequency of studies by disease type.

The general characteristics of included studies were summarised in Table 2. Among the included studies, 35 articles (42%) were published between 2019 and 2021, while 49 articles (58%) were published between 1989 and 2018. Strikingly, there was an increase in economic evaluations starting from 2014 (Fig. 2). About 62% (n = 52) were cost-effectiveness analyses, 31% (n = 26) were cost-utility analyses, 5% (n = 4) were cost-minimisation analyses, and the rest (2%, n = 2) were cost-benefit analyses. More than half of the first authors (57%, n = 48) were affiliated with Indonesian institutions and a third (32%, n = 27) of the first authors were affiliated with foreign institutions, excluding those affiliated with both institutions.

**Table 2:** General characteristics of included studies.

Items	Category	Frequency
Number of included studies		84 (100%)
Year published	Up to 2018	49 (58%)
	2019–2021	35 (42%)
Study start date^a^	up to 2018	57 (68%)
	2019–2021	27 (32%)
Study type	Cost-effectiveness	52 (62%)
	Cost-utility	26 (31%)
	Cost-minimization	4 (5%)
	Cost-benefit	2 (2%)
First author affiliation	Indonesian	48 (57%)
	Foreign	27 (32%)
	Mixed	9 (11%)
Funding	Indonesian government	18 (21%)
	Non-Indonesian government	7 (8%)
	Private (not-for-profit)	8 (10%)
	Private (for profit)	8 (10%)
	Mixed funding	13 (15%)
	No funding	3 (4%)
	Not stated	27 (32%)
Publication type	International	77 (92%)
	Local	7 (8%)
Language	English	81 (96%)
	Indonesian	3 (4%)
Disease type	Respiratory	14 (17%)
	Cardiovascular	11 (13%)
	Mosquito-borne	10 (12%)
	Cancer	8 (10%)
	Reproductive, maternal, and neonatal health	8 (10%)
	Diabetes	7 (8%)
	Digestive system	6 (7%)
	Typhoid fever	3 (4%)
	Kidney	3 (4%)
	Blood disorders	2 (2%)
	Cataract	2 (2%)
	Hepatitis	2 (2%)
	Rabies	2 (2%)
	Others	5 (6%)
	Not applicable^b^	1 (1%)
Technology type^c^	Drugs	26 (31%)
	Biological matter	22 (26%)
	Devices	5 (6%)
	Medical/surgical procedure	6 (7%)
	Support systems	17 (20%)
	Organizational and managerial system	3 (4%)
	More than 1 type	5 (6%)
Study objective	Inform decision-making at the national level	41 (49%)
	Inform decision-making at sub-national level (incl. healthcare provider setting)	14 (17%)
	Unclear objective^d^	26 (31%)
	Not mentioned	3 (4%)
Perspective	Societal	9 (11%)
	Provider	21 (25%)
	Patient	6 (7%)
	Health system	7 (8%)
	Payer	16 (19%)
	Others	1 (1%)
	Unclear	2 (2%)
	More than 1 perspective	22 (26%)
Comparator	Standard therapy/most commonly used	40 (48%)
	Most likely to be replaced	2 (2%)
	All relevant alternatives	19 (23%)
	No treatment/do nothing	12 (14%)
	Others	11 (13%)
Discount rate	With discount rate	46 (55%)
	No discount rate	2 (2%)
	Not applicable	36 (43%)
Outcome	QALY	17 (20%)
	DALY	14 (17%)
	Clinical outcomes	15 (18%)
	Life years saved/lost	8 (10%)
	Case averted/detected	5 (6%)
	Deaths averted	3 (4%)
	Others	11 (13%)
	More than 1 outcome type	11 (13%)
Study design	Study-based	41 (49%)
	Decision tree	15 (18%)
	Markov	16 (19%)
	Decision tree and Markov	1 (1%)
	Others	11 (13%)
Cost input	Direct cost	62 (74%)
	Direct and indirect cost	22 (26%)
Sensitivity analysis	Deterministic	38 (45%)
	Probabilistic	9 (11%)
	Deterministic and probabilistic	17 (20%)
	No sensitivity analysis	20 (24%)
Conflict of interest statement	Reported	62 (74%)
	Not reported	22 (26%)
Discussion on equity	Available	1 (1%)
	Not available	83 (99%)
Budget impact analysis	Available	8 (10%)
	Not available	76 (90%)
Guideline cited^e^	InaHTAC HTA guideline	2 (2%)
	MOH pharmacoeconomic guideline	11 (13%)
	WHO guidelines^f^	19 (23%)
	No guideline cited	52 (62%)

Note: ‘More than one type’ means the study reported more than one category stated in the items. ‘Other’ means that study reported other categories that has not been stated.

a Studies are assumed to take one year before the publication year, otherwise stated.

b One study assessed research and development costs in general, not applicable to any disease.

c Technology types classification is based on the guideline's classification.^11^

d Unclear objective means studies report their objective in a general context but no explanation in relevance to health policy.

e Guidelines that were referred to overall methodology recommendations were counted; guidelines that were referred to for subsection(s) of methodology were not counted.

f WHO guide to cost-effectiveness analysis and WHO guide for standardization of economic evaluations of immunization programmes.^23^^,^^24^

The most identified funding source was the Indonesian government, which accounted for at least 21% (n = 18) of all studies, excluding studies with mixed funding sources. Within the mixed funding source, the Indonesian government funded five (38%) studies together with other institutions. Funding sources were not mentioned in 32% (n = 27) of all studies. Most included studies (92%, n = 77) were published in international journals and the most used language was English (96%, n = 81).

Respiratory diseases (17%, n = 14) were the most studied condition, followed by cardiovascular diseases (13%, n = 11) and mosquito-borne diseases (12%, n = 10), respectively. The earliest economic evaluations evaluated respiratory disease and reproductive, maternal, and neonatal health, as seen in Fig. 2. Across all studies, evaluation of drugs (31%, n = 26), biological matter (26%, n = 22), and support systems (i.e., counselling programs) (20%, n = 17) were the most common technology type identified.

Forty-one studies (49%) reported their objectives as informing decision-making at the national level while 14 studies (17%) reported their objectives as informing decision-making at the sub-national level. The provider (25%, n = 21) and payer (19% n = 16) were the most common single perspectives. Twenty-two studies (26%, n = 22) used more than one perspective. The most used comparator type was standard therapy or the most used therapy (48%, n = 40), followed by all relevant alternatives (23%, n = 19). Forty-eight studies (57%) were eligible for discounting, of which two studies (2%) did not report the discount rate used. Regarding outcomes, 13% (n = 11) of the studies used more than one outcome type, while the rest used a single outcome type. Quality-adjusted life years (QALY) was used in at least 20% (n = 17) of the included studies, followed by clinical outcomes (18%, n = 15) and disability-adjusted life years (DALY) (17%, n = 14), excluding those with more than one outcome type.

Economic evaluations using clinical studies accounted for 49% (n = 41) of all studies. For model-based studies, the decision tree model was used in 18% (n = 15) of all studies and the Markov model was used in 19% (n = 16) of all studies, while both the decision tree and Markov model were used in 1% (n = 1) of all studies. As for cost input, the direct cost was the primary cost input (74%, n = 62), while the rest (26%, n = 22) included direct and indirect costs. Deterministic sensitivity analyses were performed in 45% (n = 38) of all studies, probabilistic analyses were performed in 11% (n = 9) of all studies, both analyses were performed in 20% (n = 17) of all studies, and no sensitivity analyses were performed in the remaining (24%, n = 20).

Conflict of interest statements were reported in 74% (n = 62) of the studies with 10 of those (16%) declaring a potential conflict of interest. One (1%) study briefly discussed equity, and 10% (n = 8) of the studies analysed budget impact using government and provider perspectives. Of all studies, only two (2%) studies cited the guideline and The Ministry of Health pharmacoeconomic guideline was cited in 13% (n = 11) of all studies.

### Methodology and reporting adherence

Results are summarised in Tables 3 and 4. Out of 84 studies, only 17 (20%) studies mentioned the study start date. No statistically significant difference (P > 0.05) was observed in the proportion of pre-2018 and post-2018 studies which followed the guideline's methodology recommendations, except for the outcome choice (P = 0.04) (Table 3). A significantly higher proportion of studies in post-2018 used the recommended outcomes by the guideline compared to studies in pre-2018. For reporting standards, the mean rank of post-2018 studies was significantly higher than pre-2018 studies (P = 0.01), indicating that studies have improved their reports according to the reporting standard recommendations by the guideline.

**Table 3:** Methodology and reporting adherence.

Methodology and reporting recommendations	Frequency of studies following recommendations^a^		P-value
	Pre-2018	Post-2018	
Use societal and/or provider perspective	33/57 (58%)	18/27 (67%)	0.44
Most used interventions are used as comparators	28/57 (49%)	12/27 (44%)	0.69
Same time horizon for both cost and outcome	54/57 (95%)	25/27 (93%)	0.66
Discount rate at 3% for both cost and outcome	28/32 (88%)	15/16 (94%)	0.65
Final clinical endpoint of life-years gained and/or non-clinical outcome of QALY/DALY	29/57 (51%)	20/27 (74%)	0.04
Use EQ-5D for utility measurement, according to the Indonesian context	2/14 (14%)	2/12 (17%)	1.00
Use a decision tree and/or Markov model, depends on natural disease progression	15/24 (64%)	17/19 (84%)	0.08
Use sensitivity analysis	44/57 (77%)	20/27 (74%)	0.75
Compare the ICER value to recommended WTP threshold^b^	27/55 (49%)	17/27 (63%)	0.24
Budget impact analysis	3/57 (5%)	5/27 (19%)	0.10
Clear and accurate reporting according to CHEERS^c^	0.79	0.87	0.01

a Where applicable.

b The guideline recommends using the WTP threshold of one to three times GDP per capita.^11^

c Mean rank of all studies, based on 2013 CHEERS.^12^

**Table 4:** Frequency studies following CHEERS checklist items.^12^

Items	Description	Frequency of studies following recommendations^a^	
		Pre-2018	Post-2018
1: Title	Identify the study as an economic evaluation or use more specific terms such as “cost-effectiveness analysis”, and describe the interventions compared	56/57 (98%)	27/27 (100%)
2: Abstract	Provide a structured summary of objectives, perspective, setting, methods (including study design and inputs), results (including base case and uncertainty analyses), and conclusions	11/57 (19%)	10/27 (37%)
3: Background and objectives	Provide an explicit statement of the broader context for the study. Present the study question and its relevance for health policy or practice decisions.	55/57 (96%)	26/27 (96%)
4: Target population and subgroups	Describe characteristics of the base case population and subgroups analyzed, including why they were chosen.	57/57 (100%)	27/27 (100%)
5: Setting and location	State relevant aspects of the system(s) in which the decision(s) need(s) to be made.	55/57 (96%)	27/27 (100%)
6: Study perspectives	Describe the perspective of the study and relate this to the costs being evaluated	42/57 (74%)	23/27 (85%)
7: Comparators	Describe the interventions or strategies being compared and state why they were chosen.	51/57 (89%)	26/27 (96%)
8: Time horizon	State the time horizon(s) over which costs and consequences are being evaluated and say why appropriate.	31/57 (54%)	22/27 (81%)
9: Discount rate	Report the choice of discount rate(s) used for costs and outcomes and say why appropriate.	15/32 (47%)	8/16 (50%)
10: Choice of health outcomes	Describe what outcomes were used as the measure(s) of benefit in the evaluation and their relevance for the type of analysis performed.	51/57 (89%)	24/27 (89%)
11a: Measurement of effectiveness	Single study-based estimates: Describe fully the design features of the single effectiveness study and why the single study was a sufficient source of clinical effectiveness data.	17/33 (52%)	6/12 (50%)
11b: Measurement of effectiveness	Synthesis-based estimates: Describe fully the methods used for identification of included studies and synthesis of clinical effectiveness data.	20/22 (91%)	15/15 (100%)
12: Measurement and valuation of preference based outcomes	If applicable, describe the population and methods used to elicit preferences for outcomes.	10/13 (77%)	10/12 (83%)
13a: Estimating resources and cost	Single study-based economic evaluation: Describe approaches used to estimate resource use associated with the alternative interventions. Describe primary or secondary research methods for valuing each resource item in terms of its unit cost. Describe any adjustments made to approximate to opportunity costs	19/33 (58%)	4/8 (50%)
13b: Estimating resources and cost	Model-based economic evaluation: Describe approaches and data sources used to estimate resource use associated with model health states. Describe primary or secondary research methods for valuing each resource item in terms of its unit cost. Describe any adjustments made to approximate to opportunity costs	23/25 (92%)	18/19 (95%)
14: Currency, price date, and conversion	Report the dates of the estimated resource quantities and unit costs. Describe methods for adjusting estimated unit costs to the year of reported costs if necessary. Describe methods for converting costs into a common currency base and the exchange rate.	32/57 (56%)	19/27 (70%)
15: Choice of Model	Describe and give reasons for the specific type of decision-analytical model used. Providing a figure to show model structure is strongly recommended.	20/24 (83%)	19/19 (100%)
16: Assumptions	Describe all structural or other assumptions underpinning the decision-analytical model.	24/24 (100%)	18/19 (95%)
17: Analytical methods	Describe all analytical methods supporting the evaluation. This could include methods for dealing with skewed, missing, or censored data; extrapolation methods; methods for pooling data; approaches to validate or make adjustments (such as half cycle corrections) to a model; and methods for handling population heterogeneity and uncertainty.	30/57 (53%)	19/27 (70%)
18: Study parameters	Report the values, ranges, references, and, if used, probability distributions for all parameters. Report reasons or sources for distributions used to represent uncertainty where appropriate. Providing a table to show the input values is strongly recommended.	50/57 (88%)	27/27 (100%)
19: Incremental costs and outcomes	For each intervention, report mean values for the main categories of estimated costs and outcomes of interest, as well as mean differences between the comparator groups. If applicable, report incremental cost-effectiveness ratios.	48/57 (84%)	25/27 (93%)
20a: Characterising uncertainty	Single study-based economic evaluation: Describe the effects of sampling uncertainty for the estimated incremental cost and incremental effectiveness parameters, together with the impact of methodological assumptions (such as discount rate, study perspective)	18/33 (55%)	2/9 (22%)
20b: Characterising uncertainty	Model-based economic evaluation: Describe the effects on the results of uncertainty for all input parameters, and uncertainty related to the structure of the model and assumptions.	24/26 (92%)	17/18 (94%)
21: Characterising heterogenity	If applicable, report differences in costs, outcomes, or cost-effectiveness that can be explained by variations between subgroups of patients with different baseline characteristics or other observed variability in effects that are not reducible by more information	2/11 (18%)	2/11 (18%)
22: Study findings, limitations, generalizability, and current knowledge	Summarise key study findings and describe how they support the conclusions reached. Discuss limitations and the generalizability of the findings and how the findings fit with current knowledge	45/57 (79%)	23/27 (85%)
23: Source of funding	Describe how the study was funded and the role of the funder in the identification, design, conduct, and reporting of the analysis. Describe other non-monetary sources of support	35/57 (61%)	25/27 (93%)
24: CoI	Describe any potential for conflict of interest of study contributors in accordance with journal policy. In the absence of a journal policy, we recommend authors comply with International Committee of Medical Journal Editors recommendations	37/57 (65%)	25/27 (93%)

a Where applicable.

Comparator, instrument choice for utility measurement, and budget impact analysis were items with less than 50% of studies following the methodology recommendations, even after the guideline dissemination. Most used interventions were applied as comparators in almost half of all studies. The guideline recommends that the comparisons between interventions follow the clinical context of the studied cases and the reasons for their relevance must support the selection of the comparisons. This has been reported well in all of the studies post-2018, except for one study comparing ‘treatments’ for diabetes patients with different complications without defining the treatments.^24^ Without a proper explanation of the intervention, the study may be potentially misleading.

For utility measurement, the proportion of studies using EQ-5D adapted to the Indonesian value set was low (14% (n = 2) and 17% (n = 2) in pre-2018 and post-2018 studies, respectively). Apart from EQ-5D, some studies used specific utility measurements for certain conditions, such as SF-36. The budget impact was only analysed in less than ten studies in the pre- and post-2018 period.

Perspective, time horizon, discount rate, modelling, sensitivity analysis, and comparison to the WTP threshold (willingness-to-pay threshold) were items with more than 50% of studies following the recommendation. Apart from societal and provider perspectives, the payer perspective was another common perspective used, as researchers were trying to evaluate the value of health technologies for BPJS, the payer of JKN.

The time horizon specified was the same for both cost and outcome. A high frequency of studies followed the guideline recommendation to use a 3% discount rate, but the rationale for using such a rate was not explained in half of the pre-2018 and post-2018 studies.

The results showed a significant increase (P = 0.04) in the proportion of post-2018 studies following the guideline recommendation of outcome choice compared to pre-2018 studies. About 90% of studies pre-2018 (n = 51) and post-2018 (n = 24) followed the reporting standard by justifying the outcome choice. CHEERS categorised two types of effectiveness measures, using single study-based and synthesis-based estimates. The result was somehow recorded that a lower proportion of economic evaluations using single study-based estimates (52% (n = 17) and 50% (n = 6) in pre-2018 and post-2018 periods, respectively) reported the design features or methods to generate effectiveness compared to synthesis-based estimates (91% (n = 20) and 100% (n = 15) in pre-2018 and post-2018, respectively).

Similarly, the CHEERS checklist separates single study-based and model-based evaluation for sensitivity analysis. Sensitivity analysis was sufficiently done in most model-based evaluations [92% (n-24) and 94% (n = 17) in pre-2018 and post-2018 periods, respectively], in contrast with single study-based evaluations (55% (n = 18) and 22% (n = 2) in pre-2018 and post-2018 periods, respectively).

The recommendation for the WTP threshold is one to three times the gross domestic product per capita.^11^ Some studies defined the threshold using other sources. For example, the study by Mahendradhata and colleagues set a threshold based on an estimated marginal cost of the National Tuberculosis Control Program to assess the cost-effectiveness of referring tuberculosis suspects through private practitioners for directly observed treatment short course in Jogjakarta.^25^

The results for additional analyses were presented in Tables 5 and 6. A forward stepwise fractional logistic regression model obtained two possible predictors for methodology adherence score (involvement of foreign affiliation and study design) and two possible predictors for reporting adherence score (source of funding and study design).

**Table 5:** Relationship between study characteristics and methodology adherence.

Study characteristic		Frequency of study	Methodology Coef. (95% CI)	P-value
Involvement of foreign affiliation	No	34	Ref	
	Yes	50	0.62 (0.27–0.97)	0.00
Study design	Clinical study based	41	Ref	
	Model based	43	0.57 (0.23–0.90)	0.00

**Table 6:** Relationship between study characteristics and reporting adherence.

Study characteristic		Frequency of study	Reporting Coef. (95% CI)	P-value
Source of funding	No funding or not stated	30	Ref	
	Government	25	0.50 (0.13–0.88)	0.01
	Private	16	0.67 (0.12–1.23)	0.02
	Mix (government and private)	13	0.91 (0.36–1.46)	0.00
Study design	Clinical study based	41	Ref	
	Model based	43	0.80 (0.44–1.15)	<0.00

A higher methodology adherence (statistically significant, P = 0^.^00) was demonstrated in studies with foreign affiliation and model-based studies compared to each characteristic reference. Studies funded by the government, private, and mixed funding have significantly higher reporting adherence (P < 0.05) than those with no funding or no statement of funding. Model-based studies also have higher reporting adherence (statistically significant, P = 0.00) than clinical study-based evaluations.

### Source of evidence

**Fig. 3:**
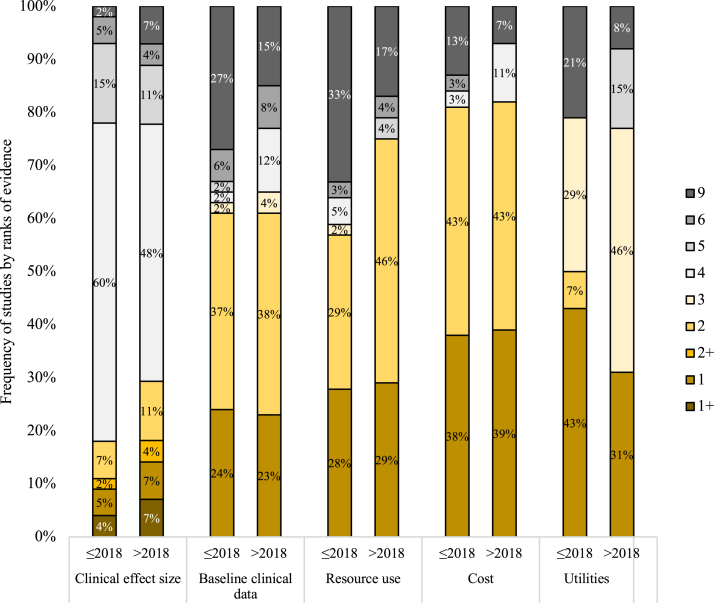
Frequency of study by ranks of evidence.

The ranks of evidence are shown in Fig. 3. The guideline recommends obtaining clinical effectiveness from systematic review and meta-analysis studies; however, the clinical effectiveness data were mainly sourced from observational studies at the regional or institutional level, with the baseline data from patients in the same jurisdiction. The utilities were mainly sourced from direct or indirect assessments, either specific to the study or using data from previous research. Similarly, most studies used primary data sources for resource use and cost, either collected or using administrative databases, as recommended by the guideline.

### Sensitivity analyses

The results are summarised in Table 7. Both sensitivity analyses yielded no statistically significant difference in study proportions (P > 0.05) following methodology recommendations in both periods, except for the type of modelling (statistically significant, P = 0.03). A higher proportion of studies in the post-dissemination period using a decision tree and/or Markov model compared to the pre-dissemination period in both sensitivity analyses. For reporting the results according to the CHEERS checklist, the difference between pre-dissemination and post-dissemination periods were changed to statistically insignificant in both scenarios (P > 0.05).

**Table 7:** Sensitivity analyses result.

Methodology and reporting recommendations	Frequency of studies following recommendations^a^		P-value	Frequency of studies following recommendations^a^		P-value
	Pre-2017	Post-2017		Pre-2016	Post-2016	
Use societal and/or provider perspective	26/44 (59%)	25/40 (63%)	0.75	23/40 (58%)	28/44 (64%)	0.57
Most used interventions used as comparators	21/44 (48%)	19/40 (48%)	0.98	20/40 (50%)	20/44 (45%)	0.68
Same time horizon for both cost and outcome	42/44 (95%)	37/40 (93%)	0.67	39/40 (98%)	40/44 (91%)	0.36
Discount rate at 3% for both cost and outcome	22/25 (88%)	21/23 (91%)	1.00	22/25 (88%)	21/23 (91%)	1.00
Final clinical endpoint of life-years gained and non-clinical outcome of QALY/DALY for outcome type	24/44 (55%)	25/40 (63%)	0.46	23/40 (58%)	26/44 (59%)	0.88
Use EQ-5D for utility measurement, according to the Indonesian context	1/11 (9%)	3/15 (20%)	1.00	1/10 (10%)	3/16 (19%)	1.00
Use a decision tree and/or Markov model, depends on natural disease progression	13/22 (59%)	19/21 (90%)	0.03	13/22 (59%)	19/21 (90%)	0.03
Use sensitivity analysis	35/44 (80%)	29/40 (73%)	0.45	33/40 (83%)	31/44 (70%)	0.20
Compare the ICER value to recommended WTP threshold^b^	21/44 (48%)	23/38 (61%)	0.25	21/40 (53%)	23/42 (55%)	0.84
Budget impact analysis	3/44 (7%)	5/40 (13%)	0.47	3/40 (8%)	5/44 (11%)	0.72
Clear and accurate reporting according to CHEERS^c^	0.80	0.85	0.11	0.82	0.83	0.57

a Where applicable.

b The guideline recommends using the WTP threshold of one to three times GDP per capita.^11^

c Mean rank of all studies, based on 2013 CHEERS.^12^

## Discussion

This study reviewed guideline adherence and source of evidence quality of health economic evaluations for Indonesia. Similar studies were available for other contexts. For example, a study by Emerson and colleagues in 2019 measured improvement in the methodological and reporting practice of cost-effectiveness studies by scoring studies’ adherence to the iDSI reference case.^26^ To our knowledge, no similar analysis for the context of Indonesia has been published.

Economic evaluation has become crucial evidence for products and services included in the JKN benefit packages.^5^^,^^14^ Therefore, appraising the methodology and reporting relevance to the local context is essential to improve future studies' usefulness in supporting health resource allocation. In addition, it can help to improve the guideline implementation plan and provide inputs for future development of the next or similar guidelines. However, we noted that not all studies under review mentioned its objective to support health policy. Yet, HTA helps inform resource allocation; therefore, with or without mentioning the study's relevance to health policy, these studies intend to support policymaking. Hence, we could not make an exclusion criterion for the study objective. We included all economic evaluations for Indonesia to ensure relevant economic evaluations were appraised. On another note, this study assessed adherence and source of evidence quality based on reported articles, indicating that failure to report relevant items will result in a lower score.

According to our search strategy, Indonesia's first health economic evaluation was published in 1989. The surge of published articles was between 2014 and 2017, the same period the guideline was issued, and the InaHTAC was commissioned. A more plausible reason for this surge was InaHTAC's collaboration with international partners because only two articles cited the guideline while eleven studies cited the pharmacoeconomic guideline by the Ministry of Health, Indonesia.^8^ The studies, which cited the pharmacoeconomic guideline, were published between 2017 and 2021. Perhaps, it takes about four to five years to disseminate a guideline in Indonesia, or there was a lack of technical capacity of researchers to follow the reference cases in the guideline. There may also be competing influences of guidelines that are more accepted in the international context, such as the WHO guide to cost-effectiveness analysis and the WHO guide for standardisation of economic evaluations of immunisation programmes.^27^^,^^28^ Moreover, among the 23 studies funded by the Indonesian government (including mix-funded studies), only two studies cited the guideline and only four studies cited the pharmacoeconomic guideline implying low uptake of guidelines by government-funded studies. Surprisingly, although a study explicitly mentioned its funding by BPJS and the Center of Financing and Health insurance, it did not cite any guidelines.^29^ Future research would help to understand the reasons for lower citations of the guidelines.

Results of the present study indicate that the guideline did not improve most of the methodology and reporting standards of the included studies. Some items in the methodology recommendations, such as perspective, time horizon, discount rate, modelling type, sensitivity analysis, and ICER (incremental cost-effectiveness ratio) comparison to WTP threshold, have been applied before the guideline was disseminated. In contrast, other items such as comparator, instrument choice for utility measurement, and budget impact analysis have not been adopted even after the guideline was issued. Moreover, there were some concerns about methodology, reporting, and source of evidence found in the included studies.

Observational studies were the source for most of the clinical effectiveness data while the guideline recommends using a systematic review of randomised controlled trials (RCTs). As there might be potential bias or confounding from observational studies, readers should ensure proper statistical techniques are used to adjust the potential bias or confounding. This suggestion should be included in future guidelines, as well as in prioritising the use of overseas RCT results over local observational data.

Sensitivity analyses were conducted less in economic evaluations using clinical studies than in model-based evaluations. The proportion of evaluations using clinical studies that conducted sensitivity analyses decreased after the guideline was published. Future studies could explore the reasons for this and consider possible solutions, as it is essential to account for the uncertainties of the input data as recommended by the guideline.

Only a few studies analysed the budget impact of the studied intervention and discussed equity implications. The absence of such analysis will complicate the readers' understanding of the intervention's affordability as budget impact analysis can provide them with a comprehensive view of new interventions (e.g., cost, effectiveness, and financial implications).^30^ The Ministry of Health Decree No. 51, 2017 has also enforced to report budget impact analysis.^14^ Furthermore, worldwide experience indicated that affordability and sustainability are important considerations when making coverage decisions.^30^ Future studies could explore the reasons to propose potential solutions in helping researchers conduct such analyses.

On that note, we have four recommendations to improve the usefulness of economic evaluations for reimbursement decision-making in Indonesia. These recommendations were based on our hypothesis that study usefulness can be enhanced by having a good study quality that is relevant to the local context.^13^

First, we recommend that the guideline be improved, at least in terms of consistency, justification, and clarity. During extraction of the guideline, we found inconsistent recommendations for perspectives used. The guideline recommends using both societal and provider perspectives on page 88, yet on page 94 it mentions using a societal perspective only. On the other hand, the guideline did not justify its recommendations, such as the discount rate used. It recommends using a 3% rate, a typical discount rate in health technology evaluation, but one study stated that using at least 5% is more appropriate for low- and middle-income countries.^31^ The guideline also lacks clarity in some of the recommendations. For example, it seems unsure in choosing the target population of whom QALY (Quality-Adjusted Life Year) should be measured. It explains the dilemma in deciding the target population and concludes that no consensus has been reached. The lack of clarity may lead to poor quality of studies, and its use for decision-making could be limited. Also, there should be a clearer intention, whether it is mandatory or recommendatory for studies to follow the guideline to be considered for reimbursement decision-making. Perhaps, the recommendation of using the guideline could come from the HTA appraisal committee or even be mandated in a regulation. Moreover, the future guideline should provide practical recommendations for conducting evaluations alongside a clinical study, given the lower adherence level compared to model-based studies.

Second, we found that the guideline has been disseminated through partner websites, presented in a national forum, and mentioned in a published article.^6^^,^^8^^,^^32^ However, considering the low uptake, we recommend that the government develop better strategies for guideline dissemination. For example, the HTA agency of Thailand disseminated their local guideline through an academic journal and a book, as well as made it available as hard and soft copies (free online version).^33^ Training and workshops were also provided to support the guideline implementation. The Equator Network recommended creating a dedicated website for the guideline and supporting resources and writing online articles about the guideline.^34^

Third, considering the high frequency of studies not stating the funding source (33%, n = 27) and conflict of interest (26%, n = 22), we recommend that international and local journal editors suggest studies declare these two crucial components; therefore, readers could be aware of potential bias.

Fourth, the present study has shown that higher methodology adherence was generated by studies with the author(s) affiliated with foreign institutions. Hence, we recommend that researchers and funders collaborate with more foreign institutions that have produced high-quality economic evaluations in the short term while increasing the capacity of Indonesian researchers. To develop local capacity, strategies such as providing adequate funding and incentives, availability of training, and quality assurance supported by political commitment could be explored.^35^^,^^36^

Results from this review has some limitations. First, we only included published studies up to December 2021, due to the timeline limitation of this study. On top of that, we only use two local journals, *JMKI* and *JEKI*, in searching for local studies because they contain articles reviewed by the Indonesian Health Economics Association. Future research to re-assess studies' adherence to the guideline with a longer dissemination period and include more local journals may provide a different results. We did not conduct our search strategy within specific databases, such as the Cost-Effective Analysis Registry by Tufts, because it only archives cost-utility studies; hence, it would not affect the search results as those studies should be available in more general databases we used (PubMed, Embase, and Ovid).^37^ Also, a search in grey literature was not conducted due to the limited timeline and resources.

Second, the scoring for methodology and reporting of the included studies was uniformly weighted between different parameters. This is because there is no international literature providing a reasonable approach to making different weights.^38^ Additionally, the reporting score might not be a relevant parameter to assess adherence to the guideline, as CHEERS is a widely used instrument in economic evaluation. Nevertheless, this review should provide an estimate of the adherence of economic evaluations for Indonesia to the guideline.

This review provides some suggestions to improve the usefulness of economic evaluations for reimbursement decision-making in Indonesia. The guideline serves as an essential document for setting a standard, however, this study showed that the guideline has minimum uptake and did not improve most of the lacking methodology and reporting standards of included studies. There should be necessary action to improve the guideline and dissemination efforts. Furthermore, there should be a policy and guidance on how these studies could support the decision-making process of the JKN benefit packages.

## Contributors

KKC and YT conceptualised the study. KKC conducted the literature search. KKC planned and managed project execution. KKC and YT designed the study while DF provided inputs. KKC and DF searched the databases and journals. KKC and DF reviewed the title, abstract, and full text. KKC, DF, and ENS extracted and managed the data. YT was consulted in case of any dispute and supervised the overall process. KKC analysed the results with consultation from YW. YW provided critical inputs and validated the results for the data analysis. KKC drafted the manuscript and all authors reviewed, edited, and approved the final manuscript.

## Data sharing statement

All relevant data is available in this manuscript and Supplementary Material. Any other details will be made upon request.

## Declaration of interests

None. The Health Intervention and Technology Assessment Program (HITAP), a semi-autonomous research unit in the Ministry of Public Health, Thailand, supports evidence-informed priority-setting and decision-making for healthcare. HITAP is funded by national and international public funding agencies. HITAP is supported by ADP, which is hosted by the UNDP and funded by the Government of Japan to support strengthening the capacity for evidence-informed health decisions. It is also supported by the HSRI Thailand to conduct research on regional collaborations for infectious diseases.
