# Supplementary material for: Health economic evaluations for Indonesia: a systematic review assessing evidence quality and adherence to the Indonesian Health Technology Assessment (HTA) Guideline
Source: Lancet Reg Health Southeast Asia. 2023 Mar 31;13:100184. doi: 10.1016/j.lansea.2023.100184 (PMC10306002; doi:10.1016/j.lansea.2023.100184)
Supplement: Supplementary Material [file mmc1.docx]

**Supplementary material**

[I. Search strategy 2](#_Toc115352639)

[II. Methodology recommendations extraction 3](#_Toc115352640)

[III. General characteristics of included studies 6](#_Toc115352641)

[IV. Methodology extraction result 11](#_Toc115352642)

[V. Reporting extraction result 15](#_Toc115352643)

[VI. Source of evidence extraction result 27](#_Toc115352644)

[VII. Regression analyses 33](#_Toc115352645)

# **Search strategy**

Multiple search strategies were tested using different search terms to obtain as many relevant studies as possible (Table 1). KKC and DF screened each search strategy results. Strategy k was chosen as the final search strategy because it contains a high number of studies within the inclusion criteria and the lowest number of studies within the exclusion criteria.

**Table1. Search strategy**

| **Strategy** | **Search Terms** |
| --- | --- |
| a | #1 AND #4 |
| b | #1 AND #5 |
| c | #1 AND #21 AND #5 |
| d | #2 AND #4 |
| e | #2 AND #5 |
| f | #2 AND #21 AND #5 |
| g | #3 AND #4 |
| h | #3 AND #5 |
| i | #3 AND #21 AND #5 |
| j | #6 OR #7 OR #8 OR #9 OR #10 OR #11 OR #12 OR #13 OR #14 OR #15 OR #16 OR #17 OR #18 OR #19 OR #20 OR #5 |
| k | (#6 OR #7 OR #8 OR #9 OR #10 OR #11 OR #12 OR #13 OR #14 OR #15 OR #16 OR #17 OR #18 OR #19 OR #20 OR #5) AND #1 |
| l | (#6 OR #7 OR #8 OR #9 OR #10 OR #11 OR #12 OR #13 OR #14 OR #15 OR #16 OR #17 OR #18 OR #19 OR #20 OR #5) AND #2 |
| m | ('Indonesia') AND "Costs and Cost Analysis"[Mesh] |
| n | ('Southeast Asia') AND "Costs and Cost Analysis"[Mesh] |
| o | 'Indonesia' AND "cost-benefit analysis"[MeSH Terms] |
| p | 'Southeast Asia' AND "cost-benefit analysis"[MeSH Terms] |
| q | 'Asia' AND "cost-benefit analysis"[MeSH Terms] |

**Table 2. Search terms**

| **Search Term Number** | **Search Terms** |
| --- | --- |
| #1 | Indonesia |
| #2 | Southeast Asia |
| #3 | Asia |
| #4 | Health Economics |
| #5 | Economic Evaluation |
| #6 | Cost |
| #7 | Cost Analysis |
| #8 | Cost Savings |
| #9 | Cost of Illness |
| #10 | Cost Benefit |
| #11 | Cost-benefit |
| #12 | Cost Utility |
| #13 | Cost-utility |
| #14 | Cost Effectiveness |
| #15 | Cost-effectiveness |
| #16 | Cost Minimisation |
| #17 | Cost-minimisation |
| #18 | Cost Minimization |
| #19 | Cost-minimization |
| #20 | Economic Analysis |
| #21 | Health |

# **Methodology recommendations extraction**

The guideline outlines methodology recommendations in six steps of conducting economic evaluation and a budget impact analysis section as seen in Table 3.^1^ Methodology recommendations are further categorized into two, ‘methodology specification’ and ‘methodology guidance’. Methodology specification refers to a key point (or recommendation) that is specific enough to be measured across studies (e.g., the discount rate at 3%) and methodology guideline refers to a recommendation that guides researchers in conducting methodology aspects (e.g., how to formulate research questions). Only methodology specification is included in the methodology adherence assessment. In Table 3, the aspects and recommendations are taken verbatim from the guideline while the category and its rationale are also laid out.

**Table 3. Indonesia HTA guideline methodology recommendations**

| **Elements** | **Key Points (Recommendations)** | **Category** | **Notes** |
| --- | --- | --- | --- |
| Step 1: Determine the direction, scope, and study protocol | | | |
| Literature review | All relevant articles should be critically appraised using standard methods, and combined systematically with credibility and transparency | Methodology guidance | Omitted from this study; unable to obtain information in the study as the key point does not suggest to report in the article |
|  | The systematic review (SR) results help researchers to develop theoretical and conceptual frameworks for economic evaluation through modeling | Methodology guidance | Information extracted merged to the source of evidence quality extraction grid |
|  | The SR results are needed to obtain the parameter values to be used in modeling in the economic evaluation | Methodology guidance | Information extracted merged to the source of evidence quality extraction grid |
| Research questions | Formulate a structured study protocol | Methodology guidance | Omitted from this study; unable to obtain information in the study as the key point does not suggest to report in the article |
|  | Specified and well-defined research questions are the important first step | Methodology guidance | Information extracted merged to the reporting parameters extraction grid |
|  | Information related to the disease and the type of economic evaluation should be clearly stated | Methodology guidance | Information extracted merged to the reporting parameters extraction grid |
|  | Health technologies (medicines, medical devices, or others) assessed should be mentioned | Methodology guidance | Information extracted merged to the reporting parameters extraction grid |
| Target population | Determine the target population and benefits in order to develop the appropriate assessment | Methodology guidance | Information extracted merged to the reporting parameters extraction grid |
|  | Inclusion and exclusion criteria should be clearly defined | Methodology guidance | Information extracted merged to the reporting parameters extraction grid |
|  | Explain the process of data collection and analysis for a transparent and credible HTA | Methodology guidance | Information extracted merged to the reporting parameters extraction grid |
| Comparison | The interventions/health technology comparison should be clearly described | Methodology guidance | Information extracted merged to the reporting parameters extraction grid |
|  | Standards of comparison are determined based on interventions which are most often used to treat a disease, before any new technology was available for assessment | Methodology specification | - |
| Choosing the type of economic evaluation in HTA | The recommended evaluation for HTA is the CUA*, which can be compared to the threshold value | Methodology specification | Merged with the types of outcomes |
|  | Economic evaluation studies are carried out according to the steps in the cost-effectiveness analysis of CUA | - | Omitted for this study; the statement seems to be leading to the guideline itself |
|  | HTA in Indonesia uses both societal and provider perspectives | Methodology specification | - |
| Step 2: Data collection | | | |
| Efficacy, safety, and effectiveness | Gather data related to efficacy, safety, and effectiveness | Methodology guidance | The guideline mentioned safety information was only intended to register a drug for marketing authorization, therefore we omit safety aspect for this study. The rest were merged to the reporting parameters extraction grid. |
|  | SR results should provide a strong level of evidence | Methodology guidance | Information extracted merged to the source of evidence quality extraction grid |
|  | If the resources or SR are limited, an explanation will be required. As such, the limitations may impact the model’s result | Methodology guidance | Information extracted merged to the reporting parameters extraction grid |
| Time horizon | Specify the time horizon | Methodology guidance | Information extracted merged to the reporting parameters extraction grid |
|  | The time horizon should be similar both for cost and outcome | Methodology specification | - |
|  | Use a long time horizon, supported by justifications | Methodology guidance | Information extracted merged to the reporting parameters extraction grid |
| Cost | For HTA Indonesia, the economic evaluation uses a societal perspective | Methodology specification | - |
|  | Cost identification and calculation must be conducted in a systematic and transparent way, using primary data | Methodology guidance | Information extracted merged to the source of evidence quality extraction grid |
|  | Adjust future cost to present values by discounting | Methodology specification | Merged to discounting section |
| Types of outcomes | The final form of a clinical outcome is life years gained and the final form of a non-clinical outcome is QALY/DALY | Methodology specification** | - |
|  | Data sources for clinical outcomes are obtained from a SR, gathered from Indonesian and international sources | Methodology guidance | Information extracted merged to the source of evidence quality extraction grid |
|  | To obtain QALY, a utility measurement using EQ-5D is collected as primary data, according to an Indonesian context | Methodology specification | - |
| Discounting | Cost and outcome should be adjusted over time periods, but calculated as present value | Methodology guidance | Information extracted merged to the reporting parameters extraction grid |
|  | Specify a discount rate of 3% for both cost and outcome | Methodology specification | - |
| Steps 3: Building a model | | | |
| Building a model | For HTA in Indonesia, a decision tree and/or Markov model are the recommended analytic models to be applied. The preferred model depends on natural disease progression. | Methodology specification | - |
|  | The model should be described according to clinical practices in the real world. Most importantly, a model should be developed in collaboration with clinicians, in order to gain an understanding of health/disease progression (expert opinion/ad hoc panel). | Methodology guidance | Information extracted merged to the reporting parameters extraction grid |
|  | Parameters for modeling should be appropriate, with good data | Methodology guidance | Information extracted merged to the reporting parameters and source of evidence quality extraction grid |
|  | Limitations of the model should be systematically reported | Methodology guidance | Information extracted merged to the reporting parameters extraction grid |
| Step 4: Sensitivity analysis (SA) | | | |
| Sensitivity analysis | A sensitivity analysis is used to explain uncertainties in the measurement parameters | Methodology guidance | Information extracted merged to the reporting parameters extraction grid |
|  | Choose the appropriate method of sensitivity analysis: one-way, multi-way, or probabilistic | Methodology specification | - |
| Step 5: Result interpretation | | | |
| Result interpretation | The result interpretation describes if the research purposes were achieved and if the proposed health technology was proven to be cost-effective | Methodology guidance | Information extracted merged to the reporting parameters extraction grid |
|  | The comparison of the ICER value to the threshold allows the decision maker to determine if the proposed intervention has “value for money.” | Methodology specification | - |
|  | The SA result is necessary for describing parameter uncertainty in the model. | Methodology guidance | Information extracted merged to the reporting parameters extraction grid |
|  | The study results must be interpreted carefully with regards to the study limitations. | Methodology guidance | Information extracted merged to the reporting parameters extraction grid |
| Step 6: Reporting | | | |
| Reporting | Clear and accurate reporting of PTK study results is a reflection of the quality and transparency of the study | Methodology guidance | Information extracted merged to the reporting parameters extraction grid |
|  | All components of the study must be reported, but important information should remain the focus. | Methodology guidance | Information extracted merged to the reporting parameters extraction grid |
| Budget Impact Analysis | | | |
| Budget Impact Analysis | HTA requires budget impact analysis (BIA) after the economic evaluation is performed | Methodology specification | - |
|  | BIA should also be determined even if the health technology is found to be not cost-effective, but included in the JKN benefit package, because it is needed by the community | Methodology specification | - |

*CUA: cost-utility analysis

** The authors used and/or in the data extraction form, “The final form of a clinical outcome is life years gained and/or the final form of a non-clinical outcome is QALY/DALY” because the guideline recommends using CUA. In the explanation of CUA (page 80) the guideline only describes the outcomes as utility in the quality-adjusted life years (QALY) unit.

# **General characteristics of included studies**

**Table 4. General characteristics of included studies**

| **Reference no.** | **First author affiliation** | **Involvement of foreign affiliation** | **Publication Type** | **Type of Funders** | **Language** | **Disease** | **Type of technology^a^** | **Study design** |
| --- | --- | --- | --- | --- | --- | --- | --- | --- |
| ^2^ | Indonesia | N | Local | Not stated | Indonesian | Cardiovascular disease | 1 | Clinical study-based |
| ^3^ | Indonesia | N | Local | Not stated | Indonesian | Others | 7 | Clinical study-based |
| ^4^ | Indonesia | N | Local | Not stated | English | Kidney | 4 | Clinical study-based |
| ^5^ | Indonesia | N | Local | Not stated | Indonesian | Cardiovascular disease | 1 | Clinical study-based |
| ^6^ | Mix | Y | International | Mix | English | Respiratory disease | 2 | Model-based |
| ^7^ | Foreign | Y | International | No funder | English | Reproductive, maternal, neonatal | 5 | Model-based |
| ^8^ | Indonesia | Y | International | No funder | English | Cataract | 4 | Clinical study-based |
| ^9^ | Foreign | Y | International | Private NFP | English | Mosquito-borne | 2 | Model-based |
| ^10^ | Indonesia | Y | International | Indo gov | English | Mosquito-borne | 2,5 | Model-based |
| ^11^ | Foreign | Y | International | Not stated | English | Respiratory disease | 2 | Model-based |
| ^12^ | Indonesia | N | International | Indo gov | English | Mosquito-borne | 2,5 | Model-based |
| ^13^ | Foreign | Y | International | Foreign gov | English | Reproductive, maternal, neonatal | 6 | Model-based |
| ^14^ | Foreign | Y | International | No funder | English | Others | 4,5 | Model-based |
| ^15^ | Indonesia | Y | International | Indo gov | English | Cancer | 2 | Model-based |
| ^16^ | Indonesia | Y | International | Mix | English | Cardiovascular disease | 1 | Model-based |
| ^17^ | Foreign | Y | International | Mix | English | Hepatitis | 6 | Model-based |
| ^18^ | Indonesia | N | International | Private NFP | English | Respiratory disease | 2 | Model-based |
| ^19^ | Foreign | Y | International | Mix | English | Mosquito-borne | 1 | Model-based |
| ^20^ | Foreign | Y | International | Mix | English | Mosquito-borne | 1 | Model-based |
| ^21^ | Indonesia | N | International | Not stated | English | Cardiovascular disease | 1 | Clinical study-based |
| ^22^ | Foreign | Y | Local | Private NFP | English | Reproductive, maternal, neonatal | 5 | Model-based |
| ^23^ | Indonesia | N | International | Private FP | English | Cancer | 2 | Model-based |
| ^24^ | Foreign | Y | International | Not stated | English | Cancer | 1 | Model-based |
| ^25^ | Mix | Y | International | Not stated | English | Hepatitis | 2 | Model-based |
| ^26^ | Mix | Y | International | Not stated | English | Digestive system disease | 2 | Model-based |
| ^27^ | Indonesia | N | International | Indo gov | English | Reproductive, maternal, neonatal | 3 | Model-based |
| ^28^ | Mix | Y | International | Indo gov | English | Rabies | 2 | Model-based |
| ^29^ | Mix | Y | International | Indo gov | English | Cancer | 2 | Model-based |
| ^30^ | Indonesia | Y | International | Foreign gov | English | Cardiovascular disease | 1 | Model-based |
| ^31^ | Foreign | Y | International | Private NFP | English | Digestive system disease | 2 | Model-based |
| ^32^ | Indonesia | N | International | Indo gov | English | Cancer | 3 | Clinical study-based |
| ^33^ | Mix | Y | International | Private FP | English | Digestive system disease | 5 | Model-based |
| ^34^ | Foreign | Y | International | Private FP | English | Diabetes | 2 | Model-based |
| ^35^ | Mix | Y | International | Indo gov | English | Rabies | 2 | Model-based |
| ^36^ | Foreign | Y | International | Mix | English | Cardiovascular disease | 5 | Model-based |
| ^37^ | Indonesia | Y | International | Mix | English | Kidney | 3 | Model-based |
| ^38^ | Foreign | Y | International | Private FP | English | Respiratory disease | 5 | Model-based |
| ^39^ | Indonesia | N | International | Indo gov | English | Respiratory disease | 1,2 | Clinical study-based |
| ^40^ | Foreign | Y | International | Private FP | English | Diabetes | 2 | Model-based |
| ^41^ | Foreign | Y | International | Private FP | English | Diabetes | 2 | Model-based |
| ^42^ | Foreign | Y | International | Foreign gov | English | Mosquito-borne | 2 | Model-based |
| ^43^ | Mix | Y | International | Foreign gov | English | Reproductive, maternal, neonatal | 1 | Model-based |
| ^44^ | Foreign | Y | International | Mix | English | Respiratory disease | 2 | Clinical study-based |
| ^45^ | Foreign | Y | International | Not stated | English | Typhoid | 2 | Clinical study-based |
| ^46^ | Indonesia | N | International | Not stated | English | Cancer | 3 | Model-based |
| ^47^ | Foreign | Y | International | Private NFP | English | Mosquito-borne | 5 | Clinical study-based |
| ^48^ | Foreign | Y | International | Mix | English | Respiratory disease | 5 | Model-based |
| ^49^ | Indonesia | Y | International | Mix | English | Respiratory disease | 5 | Clinical study-based |
| ^50^ | Foreign | Y | International | Mix | English | Reproductive, maternal, neonatal | 5 | Clinical study-based |
| ^51^ | Foreign | Y | International | Private NFP | English | Reproductive, maternal, neonatal | 4 | Model-based |
| ^52^ | Foreign | Y | International | Mix | English | Reproductive, maternal, neonatal | 5 | Clinical study-based |
| ^53^ | Indonesia | N | International | Indo gov | English | Blood disorder | 1 | Clinical study-based |
| ^54^ | Indonesia | N | International | Indo gov | English | Cancer | 1 | Model-based |
| ^55^ | Indonesia | Y | International | Not stated | English | Kidney | 5 | Clinical study-based |
| ^56^ | Indonesia | Y | International | Indo gov | English | Not applicable | 6 | Model-based |
| ^57^ | Indonesia | Y | International | Mix | English | Others | 3 | Model-based |
| ^58^ | Indonesia | Y | International | Indo gov | English | Diabetes | 1 | Clinical study-based |
| ^59^ | Mix | Y | International | Indo gov | English | Respiratory disease | 4 | Model-based |
| ^60^ | Indonesia | N | International | Foreign gov | English | Mosquito-borne | 1 | Clinical study-based |
| ^61^ | Indonesia | N | International | Not stated | English | Others | 1 | Clinical study-based |
| ^62^ | Foreign | Y | International | Private NFP | English | Respiratory disease | 5 | Model-based |
| ^63^ | Indonesia | N | International | Indo gov | English | Respiratory disease | 5 | Clinical study-based |
| ^64^ | Indonesia | N | International | Not stated | English | Digestive system disease | 1 | Clinical study-based |
| ^65^ | Indonesia | Y | International | Foreign gov | English | Cataract | 4 | Clinical study-based |
| ^66^ | Indonesia | N | International | Indo gov | English | Cardiovascular disease | 5 | Clinical study-based |
| ^67^ | Indonesia | N | International | Indo gov | English | Respiratory disease | 5 | Clinical study-based |
| ^68^ | Indonesia | N | Local | Private FP | English | Cardiovascular disease | 1 | Model-based |
| ^69^ | Indonesia | N | International | Not stated | English | Typhoid | 1 | Clinical study-based |
| ^70^ | Indonesia | N | International | Not stated | English | Typhoid | 1 | Clinical study-based |
| ^71^ | Indonesia | N | Local | Not stated | English | Cardiovascular disease | 1 | Clinical study-based |
| ^72^ | Indonesia | N | International | Not stated | English | Cancer | 4 | Clinical study-based |
| ^73^ | Indonesia | N | International | Not stated | English | Blood disorder | 1 | Clinical study-based |
| ^74^ | Foreign | Y | International | Private FP | English | Mosquito-borne | 2 | Clinical study-based |
| ^75^ | Indonesia | N | International | Indo gov | English | Diabetes | 2 | Clinical study-based |
| ^76^ | Indonesia | N | International | Not stated | English | Diabetes | 5 | Clinical study-based |
| ^77^ | Indonesia | N | International | Not stated | English | Cardiovascular disease | 5 | Clinical study-based |
| ^78^ | Indonesia | N | International | Not stated | English | Diabetes | 2 | Clinical study-based |
| ^79^ | Indonesia | N | International | Not stated | English | Respiratory disease | 1 | Clinical study-based |
| ^80^ | Indonesia | N | International | Not stated | English | Respiratory disease | 1 | Clinical study-based |
| ^81^ | Indonesia | Y | International | Not stated | English | Others | 1 | Clinical study-based |
| ^82^ | Indonesia | N | International | Not stated | English | Digestive system disease | 1 | Clinical study-based |
| ^83^ | Indonesia | N | International | Not stated | English | Cardiovascular disease | 1 | Clinical study-based |
| ^84^ | Indonesia | Y | International | Private NFP | English | Digestive system disease | 2 | Clinical study-based |
| ^85^ | Foreign | Y | International | Foreign gov | English | Mosquito-borne | 1 | Clinical study-based |

^a^ 1) Drugs, 2) Biological matter, 3) Devices, 4) Medical and surgical procedures, 5) Support systems (e.g., telemedicine), 6) Organizational and managerial systems, (e.g., diagnosis-related groups) 7) Others

# **Study characteristics re-categorization for fractional logistic regression**

For fractional logistic regression, some study sub-characteristics were regrouped while ‘language’ and ‘journal type’ characteristics were omitted to avoid multicollinearity issues as seen in Table 5. For diseases type, new sub-characteristics were introduced based on disability-adjusted life years.^86^

**Table 5. Study characteristics for fractional logistic regression**

| Study characteristic | Original sub-characteristics | Frequency | Notes | New sub-characteristics | Frequency |
| --- | --- | --- | --- | --- | --- |
| Study design | Clinical study based | 41 | As is | Clinical study based | 41 |
|  | Model based | 43 | As is | Model based | 43 |
| Involvement of foreign affiliation | No | 34 | As is | No | 34 |
|  | Yes | 50 | As is | Yes | 50 |
| Source of funding | Indonesian government | 18 | Merged to government | No funding or not stated | 30 |
|  | Non-Indonesian government | 7 | Merged to government | Government | 25 |
|  | Private (not-for-profit) | 8 | Merged to private | Private | 16 |
|  | Private (for profit) | 8 | Merged to private | Mix (government and private) | 13 |
|  | Mixed funding | 13 | As is |  |  |
|  | No funding | 3 | Merged to no funding or not stated |  |  |
|  | Not stated | 27 | Merged to no funding or not stated |  |  |
| First author affiliation | Indonesian | 48 | As is | Indonesian | 48 |
|  | Foreign | 27 | Merged to foreign or mix | Foreign or mix | 36 |
|  | Mixed | 9 | Merged to foreign or mix |  |  |
| Technology type | Drugs | 26 | As is; added with study from ‘more than one type’ | Drug | 27 |
|  | Biological matter | 22 | As is | Biological matter | 22 |
|  | Devices | 5 | Merged to others | Support system | 20 |
|  | Medical/surgical procedure | 6 | Merged to others | Others | 15 |
|  | Support systems | 17 | As is; added with study from ‘more than one type’ |  |  |
|  | Organizational and managerial system | 3 | Merged to others |  |  |
|  | More than 1 type | 5 | Merged to one of the types |  |  |
| Disease | Respiratory | 14 | Merged to top 10 burdens by DALY | Top 10 burdens by DALY | 43 |
|  | Cardiovascular | 11 | Merged to top 10 burdens by DALY | Non-top 10 burdens by DALY | 41 |
|  | Mosquito-borne | 10 | Merged to non-top 10 burdens by DALY |  |  |
|  | Cancer | 8 | Merged to top 10 burdens by DALY |  |  |
|  | Reproductive, maternal, and neonatal health | 8 | Merged to non-top 10 burdens by DALY |  |  |
|  | Diabetes | 7 | Merged to top 10 burdens by DALY |  |  |
|  | Digestive system | 6 | Merged to non-top 10 burdens by DALY |  |  |
|  | Typhoid fever | 3 | Merged to non-top 10 burdens by DALY |  |  |
|  | Kidney | 3 | Merged to top 10 burdens by DALY |  |  |
|  | Blood disorders | 2 | Merged to non-top 10 burdens by DALY |  |  |
|  | Cataract | 2 | Merged to non-top 10 burdens by DALY |  |  |
|  | Hepatitis | 2 | Merged to non-top 10 burdens by DALY |  |  |
|  | Rabies | 2 | Merged to non-top 10 burdens by DALY |  |  |
|  | Others | 5 | Merged to non-top 10 burdens by DALY |  |  |
|  | Not applicable^a^ | 1 | Merged to non-top 10 burdens by DALY |  |  |
| Language | English | 81 | Dominating; omitted from fractional log regression to avoid multicollinearity issue |  |  |
|  | Indonesian | 3 |  |  |  |
| Journal type | International | 77 | Dominating; omitted from fractional log regression to avoid multicollinearity issue |  |  |
|  | Local | 7 |  |  |  |

^a^ Assessed research and development costs in general, not applicable to any disease

# **Methodology extraction result**

Several methodology recommendations were extracted from the guideline.^1^ The recommendations include perspective, comparator, time horizon, discount rate, outcomes, utility, costs, willingness to pay threshold, and sensitivity analysis. A composite quality score was generated by calculating the number of ‘Y’ out of the total applicable parameters. The composite quality score was used for regression analyses.

**Table 6. Methodology extraction result**

| **Refer-ence no.** | **Perspective** | **Comparator** | **Time horizon** | **Discount rate** | **Outcome choice** | **Utility measure-ment** | **WTP threshold** | **Sensi-tivity analysis** | **Modelling Type** | **Budget Impact Analysis** | **Number of ‘Y’** | **Number of ‘N’** | **Composite quality score** |
| --- | --- | --- | --- | --- | --- | --- | --- | --- | --- | --- | --- | --- | --- |
| ^2^ | Y | Y | Y | NA | N | NA | N | N | NA | N | 3 | 4 | 0.43 |
| ^3^ | N | N | Y | N | N | NA | NA | N | NA | N | 1 | 6 | 0.14 |
| ^4^ | N | Y | Y | NA | Y | N | N | N | NA | N | 3 | 5 | 0.38 |
| ^5^ | Y | Y | Y | NA | N | NA | N | N | NA | N | 3 | 4 | 0.43 |
| ^6^ | Y | N | Y | Y | Y | N | Y | Y | Y | N | 7 | 3 | 0.70 |
| ^7^ | Y | Y | Y | Y | Y | NA | Y | Y | Y | N | 8 | 1 | 0.89 |
| ^8^ | Y | Y | Y | NA | Y | NA | Y | N | NA | N | 5 | 2 | 0.71 |
| ^9^ | Y | N | Y | Y | Y | NA | Y | Y | N | N | 6 | 3 | 0.67 |
| ^10^ | Y | Y | Y | Y | Y | N | Y | Y | Y | Y | 9 | 1 | 0.90 |
| ^11^ | Y | Y | Y | Y | Y | NA | Y | Y | Y | N | 8 | 1 | 0.89 |
| ^12^ | Y | N | Y | Y | Y | Y | Y | Y | Y | Y | 9 | 1 | 0.90 |
| ^13^ | N | Y | Y | Y | Y | NA | Y | Y | N | N | 6 | 3 | 0.67 |
| ^14^ | Y | N | Y | Y | Y | NA | Y | Y | Y | N | 7 | 2 | 0.78 |
| ^15^ | Y | Y | Y | Y | Y | Y | Y | Y | Y | Y | 10 | 0 | 1.00 |
| ^16^ | Y | Y | Y | Y | Y | N | Y | Y | Y | Y | 9 | 1 | 0.90 |
| ^17^ | Y | N | Y | Y | Y | NA | Y | Y | N | N | 6 | 3 | 0.67 |
| ^18^ | Y | Y | Y | Y | Y | N | Y | Y | Y | Y | 9 | 1 | 0.90 |
| ^19^ | Y | Y | Y | Y | Y | NA | Y | Y | Y | N | 8 | 1 | 0.89 |
| ^20^ | Y | N | Y | Y | Y | NA | Y | Y | Y | N | 7 | 2 | 0.78 |
| ^21^ | N | Y | Y | NA | N | NA | N | N | NA | N | 2 | 5 | 0.29 |
| ^22^ | N | Y | Y | Y | Y | NA | Y | Y | N | N | 6 | 3 | 0.67 |
| ^23^ | N | N | Y | Y | Y | N | Y | Y | Y | N | 6 | 4 | 0.60 |
| ^24^ | Y | N | Y | NA | Y | N | Y | Y | Y | N | 6 | 3 | 0.67 |
| ^25^ | Y | N | Y | Y | Y | N | Y | Y | Y | N | 7 | 3 | 0.70 |
| ^26^ | Y | N | Y | Y | Y | N | Y | Y | Y | Y | 8 | 2 | 0.80 |
| ^27^ | N | N | Y | NA | Y | NA | N | Y | Y | N | 4 | 4 | 0.50 |
| ^28^ | Y | N | Y | N | N | NA | N | Y | N | N | 3 | 6 | 0.33 |
| ^29^ | N | Y | Y | Y | Y | N | Y | Y | Y | N | 7 | 3 | 0.70 |
| ^30^ | N | Y | Y | Y | Y | N | Y | Y | Y | N | 7 | 3 | 0.70 |
| ^31^ | Y | N | Y | Y | Y | NA | Y | Y | N | N | 6 | 3 | 0.67 |
| ^32^ | Y | Y | Y | NA | N | NA | N | Y | NA | N | 4 | 3 | 0.57 |
| ^33^ | Y | Y | Y | Y | Y | N | Y | Y | Y | Y | 9 | 1 | 0.90 |
| ^34^ | Y | N | Y | Y | Y | N | Y | Y | Y | N | 7 | 3 | 0.70 |
| ^35^ | N | N | Y | Y | Y | NA | N | Y | N | N | 4 | 5 | 0.44 |
| ^36^ | N | Y | Y | Y | Y | NA | Y | Y | Y | N | 7 | 2 | 0.78 |
| ^37^ | Y | N | Y | Y | Y | Y | Y | Y | Y | Y | 9 | 1 | 0.90 |
| ^38^ | N | N | Y | N | N | NA | N | Y | Y | N | 3 | 6 | 0.33 |
| ^39^ | N | N | Y | NA | N | NA | N | N | NA | N | 1 | 6 | 0.14 |
| ^40^ | N | N | Y | N | Y | N | Y | Y | Y | N | 5 | 5 | 0.50 |
| ^41^ | N | N | Y | Y | Y | N | Y | Y | Y | N | 6 | 4 | 0.60 |
| ^42^ | N | N | Y | Y | Y | NA | Y | Y | N | N | 5 | 4 | 0.56 |
| ^43^ | Y | Y | Y | Y | N | NA | Y | Y | N | N | 6 | 3 | 0.67 |
| ^44^ | Y | Y | Y | Y | Y | NA | Y | Y | NA | N | 7 | 1 | 0.88 |
| ^45^ | Y | N | Y | Y | Y | NA | N | Y | NA | N | 5 | 3 | 0.63 |
| ^46^ | Y | N | Y | NA | Y | NA | N | N | Y | N | 4 | 4 | 0.50 |
| ^47^ | Y | Y | Y | Y | N | NA | N | Y | NA | N | 5 | 3 | 0.63 |
| ^48^ | Y | N | Y | Y | N | NA | N | Y | N | N | 4 | 5 | 0.44 |
| ^49^ | Y | Y | Y | Y | N | NA | N | Y | NA | N | 5 | 3 | 0.63 |
| ^50^ | Y | N | Y | NA | N | NA | N | Y | NA | N | 3 | 4 | 0.43 |
| ^51^ | Y | N | N | NA | N | NA | N | N | N | N | 1 | 7 | 0.13 |
| ^52^ | Y | Y | Y | NA | N | NA | N | Y | NA | N | 4 | 3 | 0.57 |
| ^53^ | Y | Y | Y | NA | Y | Y | Y | Y | NA | N | 7 | 1 | 0.88 |
| ^54^ | Y | Y | Y | Y | Y | N | Y | Y | Y | N | 8 | 2 | 0.80 |
| ^55^ | N | Y | Y | NA | Y | NA | N | Y | NA | N | 4 | 3 | 0.57 |
| ^56^ | N | N | Y | NA | N | NA | N | Y | Y | N | 3 | 5 | 0.38 |
| ^57^ | Y | N | Y | Y | Y | N | Y | Y | Y | N | 7 | 3 | 0.70 |
| ^58^ | Y | N | Y | Y | N | NA | NA | N | NA | N | 3 | 4 | 0.43 |
| ^59^ | N | Y | Y | Y | Y | NA | Y | Y | Y | N | 7 | 2 | 0.78 |
| ^60^ | Y | N | Y | NA | N | NA | N | N | NA | N | 2 | 5 | 0.29 |
| ^61^ | N | Y | N | NA | Y | N | N | Y | NA | N | 3 | 5 | 0.38 |
| ^62^ | N | N | N | Y | N | NA | N | Y | Y | N | 3 | 6 | 0.33 |
| ^63^ | Y | Y | N | NA | Y | N | N | Y | NA | N | 4 | 4 | 0.50 |
| ^64^ | Y | N | Y | N | N | NA | N | N | NA | N | 2 | 6 | 0.25 |
| ^65^ | N | N | Y | NA | Y | N | Y | N | NA | N | 3 | 5 | 0.38 |
| ^66^ | N | Y | N | NA | Y | N | N | N | NA | N | 2 | 6 | 0.25 |
| ^67^ | Y | N | Y | NA | N | NA | N | N | NA | N | 2 | 5 | 0.29 |
| ^68^ | N | Y | Y | Y | Y | N | Y | Y | Y | N | 7 | 3 | 0.70 |
| ^69^ | Y | N | Y | NA | N | NA | N | Y | NA | N | 3 | 4 | 0.43 |
| ^70^ | Y | N | Y | NA | N | NA | N | Y | NA | N | 3 | 4 | 0.43 |
| ^71^ | N | Y | Y | Y | N | NA | N | N | NA | N | 3 | 5 | 0.38 |
| ^72^ | Y | Y | Y | NA | N | NA | N | Y | NA | N | 4 | 3 | 0.57 |
| ^73^ | Y | Y | Y | NA | N | NA | N | Y | NA | N | 4 | 3 | 0.57 |
| ^74^ | Y | N | Y | Y | Y | NA | Y | Y | NA | N | 6 | 2 | 0.75 |
| ^75^ | N | N | Y | NA | N | NA | N | N | NA | N | 1 | 6 | 0.14 |
| ^76^ | N | N | Y | NA | N | NA | Y | Y | NA | N | 3 | 4 | 0.43 |
| ^77^ | N | N | Y | NA | N | NA | Y | Y | NA | N | 3 | 4 | 0.43 |
| ^78^ | N | N | Y | NA | N | NA | N | N | NA | N | 1 | 6 | 0.14 |
| ^79^ | N | Y | Y | NA | N | NA | N | Y | NA | N | 3 | 4 | 0.43 |
| ^80^ | N | N | Y | NA | N | NA | Y | Y | NA | N | 3 | 4 | 0.43 |
| ^81^ | Y | Y | Y | NA | Y | NA | N | Y | NA | N | 5 | 2 | 0.71 |
| ^82^ | N | Y | Y | NA | N | NA | N | N | NA | N | 2 | 5 | 0.29 |
| ^83^ | N | Y | Y | NA | N | NA | N | N | NA | N | 2 | 5 | 0.29 |
| ^84^ | Y | N | Y | Y | Y | NA | Y | Y | NA | N | 6 | 2 | 0.75 |
| ^85^ | Y | Y | Y | NA | N | NA | Y | Y | NA | N | 5 | 2 | 0.71 |

# **Reporting extraction result**

Reporting items were extracted based on twenty-four items from the CHEERS checklist.^87^ A composite quality score was generated for each study by calculating the number of ‘Y’ out of the applicable parameters.

**Table 7. Reporting extraction result**

| **Reference no.** | **1: Title** | **2: Abstract** | **3:Back-ground and objectives** | **4: Target population and subgroups** | **5: Setting and location** | **6: Study perspectives** | **7: Compa-rators** | **8: Time horizon** | **9: Discount rate** | **10: Choice of health outcomes** | **11a: Measure-ment of effectiveness** | **11b: Measure-ment of effectiveness** |
| --- | --- | --- | --- | --- | --- | --- | --- | --- | --- | --- | --- | --- |
| ^2^ | Y | Y | Y | Y | Y | Y | Y | N | N/A | Y | N | N/A |
| ^3^ | Y | N | Y | Y | Y | N | N | Y | N | Y | Y | N/A |
| ^4^ | Y | N | Y | Y | Y | Y | Y | N | N/A | Y | N/A | N/A |
| ^5^ | Y | N | Y | Y | Y | Y | Y | Y | N/A | N | N/A | N/A |
| ^6^ | Y | Y | Y | Y | Y | Y | Y | Y | Y | Y | N/A | Y |
| ^7^ | Y | Y | Y | Y | Y | Y | Y | Y | Y | Y | N/A | Y |
| ^8^ | Y | N | Y | Y | Y | N | Y | Y | N/A | Y | N | N/A |
| ^9^ | Y | N | Y | Y | Y | Y | Y | Y | Y | Y | Y | N/A |
| ^10^ | Y | Y | Y | Y | Y | Y | Y | Y | N | Y | N/A | Y |
| ^11^ | Y | Y | Y | Y | Y | Y | Y | Y | N | Y | N/A | Y |
| ^12^ | Y | N | Y | Y | Y | Y | Y | Y | Y | Y | N/A | Y |
| ^13^ | Y | Y | Y | Y | Y | Y | N | Y | Y | Y | N/A | Y |
| ^14^ | Y | N | Y | Y | Y | N | Y | Y | N | Y | N/A | Y |
| ^15^ | Y | N | Y | Y | Y | N | Y | Y | Y | Y | N/A | Y |
| ^16^ | Y | N | Y | Y | Y | Y | Y | Y | N | Y | Y | N/A |
| ^17^ | Y | Y | Y | Y | Y | Y | Y | Y | N | Y | N/A | Y |
| ^18^ | Y | Y | Y | Y | Y | Y | Y | Y | Y | Y | N/A | Y |
| ^19^ | Y | N | Y | Y | Y | Y | Y | Y | N | Y | N/A | Y |
| ^20^ | Y | Y | Y | Y | Y | Y | Y | Y | Y | Y | N/A | Y |
| ^21^ | Y | Y | Y | Y | Y | Y | Y | Y | N/A | Y | N | N/A |
| ^22^ | Y | N | Y | Y | Y | Y | Y | Y | N | Y | N/A | Y |
| ^23^ | Y | N | Y | Y | Y | Y | Y | Y | N | Y | N/A | N |
| ^24^ | Y | Y | Y | Y | Y | Y | Y | Y | N/A | Y | N/A | Y |
| ^25^ | Y | N | Y | Y | Y | Y | Y | Y | N | Y | N/A | Y |
| ^26^ | Y | Y | Y | Y | Y | Y | Y | Y | Y | Y | N/A | Y |
| ^27^ | Y | N | Y | Y | Y | Y | Y | Y | N/A | Y | Y | N/A |
| ^28^ | Y | N | Y | Y | Y | Y | N | Y | N | Y | N/A | Y |
| ^29^ | Y | N | Y | Y | Y | Y | Y | Y | Y | Y | N | Y |
| ^30^ | Y | Y | Y | Y | Y | Y | Y | Y | Y | Y | N/A | Y |
| ^31^ | Y | N | Y | Y | Y | Y | Y | Y | Y | Y | N/A | Y |
| ^32^ | Y | N | Y | Y | Y | Y | Y | Y | N/A | Y | Y | N/A |
| ^33^ | Y | Y | Y | Y | Y | Y | Y | Y | Y | Y | N/A | Y |
| ^34^ | Y | N | Y | Y | Y | Y | Y | Y | N | Y | N/A | N/A |
| ^35^ | Y | N | Y | Y | Y | Y | Y | Y | Y | Y | N/A | Y |
| ^36^ | Y | Y | Y | Y | Y | Y | Y | Y | Y | Y | Y | N/A |
| ^37^ | N | N | Y | Y | Y | Y | Y | Y | N | Y | Y | N/A |
| ^38^ | Y | N | Y | Y | Y | N | Y | Y | N | Y | N/A | Y |
| ^39^ | Y | N | Y | Y | Y | N | Y | Y | N/A | Y | Y | N/A |
| ^40^ | Y | N | Y | Y | Y | N | Y | Y | N | Y | Y | N/A |
| ^41^ | Y | N | Y | Y | Y | N | Y | Y | Y | Y | Y | N/A |
| ^42^ | Y | N | Y | Y | Y | Y | Y | Y | Y | Y | N/A | Y |
| ^43^ | Y | N | Y | Y | Y | Y | Y | Y | N | Y | N/A | N |
| ^44^ | Y | N | Y | Y | Y | Y | Y | Y | Y | Y | Y | N/A |
| ^45^ | Y | N | Y | Y | Y | Y | Y | Y | Y | Y | N/A | Y |
| ^46^ | Y | N | Y | Y | Y | Y | Y | Y | N/A | Y | Y | N/A |
| ^47^ | Y | N | Y | Y | Y | Y | Y | Y | N | Y | Y | N/A |
| ^48^ | Y | Y | Y | Y | Y | N | Y | N | N | Y | Y | N/A |
| ^49^ | Y | N | Y | Y | Y | Y | Y | N | N | Y | Y | N/A |
| ^50^ | Y | N | Y | Y | Y | Y | Y | N | N/A | Y | Y | N/A |
| ^51^ | Y | N | Y | Y | Y | Y | Y | N | N/A | Y | N/A | Y |
| ^52^ | Y | N | Y | Y | Y | N | Y | Y | N/A | Y | N/A | Y |
| ^53^ | Y | N | Y | Y | Y | Y | Y | Y | N/A | Y | Y | N/A |
| ^54^ | Y | N | Y | Y | Y | Y | Y | Y | N | Y | N/A | Y |
| ^55^ | Y | N | Y | Y | Y | Y | Y | N | N/A | Y | N | N/A |
| ^56^ | Y | Y | Y | Y | Y | Y | Y | Y | N/A | Y | N/A | Y |
| ^57^ | Y | N | Y | Y | Y | Y | Y | Y | N | Y | N/A | Y |
| ^58^ | Y | Y | Y | Y | Y | Y | Y | N | N | Y | N | N/A |
| ^59^ | Y | N | Y | Y | Y | Y | Y | Y | Y | Y | N/A | Y |
| ^60^ | Y | N | Y | Y | Y | Y | N | N | N/A | N | N | N/A |
| ^61^ | Y | N | Y | Y | Y | Y | Y | N | N/A | Y | Y | N/A |
| ^62^ | Y | N | Y | Y | Y | Y | Y | N | N | N | N/A | Y |
| ^63^ | Y | Y | N | Y | Y | Y | Y | N | N/A | Y | N | N/A |
| ^64^ | Y | N | Y | Y | Y | Y | Y | N | N | N | N | N/A |
| ^65^ | Y | N | Y | Y | Y | Y | Y | N | N/A | Y | N | N/A |
| ^66^ | Y | N | Y | Y | Y | N | Y | N | N/A | N | N | N/A |
| ^67^ | Y | Y | Y | Y | Y | Y | Y | N | N/A | Y | N | N/A |
| ^68^ | Y | Y | Y | Y | Y | Y | Y | Y | Y | Y | N/A | Y |
| ^69^ | Y | N | Y | Y | Y | N | Y | N | N/A | Y | Y | N/A |
| ^70^ | Y | N | Y | Y | Y | Y | Y | N | N/A | N | N | N/A |
| ^71^ | Y | N | Y | Y | Y | N | Y | N | N | Y | Y | N/A |
| ^72^ | Y | N | Y | Y | Y | N | Y | N | N/A | N | N | N/A |
| ^73^ | Y | N | Y | Y | Y | N | Y | N | N/A | Y | N | N/A |
| ^74^ | Y | N | Y | Y | Y | Y | Y | Y | Y | Y | N/A | Y |
| ^75^ | Y | N | Y | Y | Y | N | Y | N | N/A | Y | N | N/A |
| ^76^ | Y | N | Y | Y | Y | Y | Y | N | N/A | Y | Y | N/A |
| ^77^ | Y | N | Y | Y | Y | Y | Y | N | N/A | Y | N | N/A |
| ^78^ | Y | N | Y | Y | Y | N | N | N | N/A | Y | N | N/A |
| ^79^ | Y | N | Y | Y | Y | N | N | N | N/A | Y | Y | N/A |
| ^80^ | Y | N | Y | Y | Y | Y | Y | N | N/A | N | N | N/A |
| ^81^ | Y | N | N | Y | N | Y | Y | N | N/A | N | N | N/A |
| ^82^ | Y | N | N | Y | N | N | N | N | N/A | Y | N | N/A |
| ^83^ | Y | N | Y | Y | Y | Y | Y | N | N/A | Y | N | N/A |
| ^84^ | Y | N | Y | Y | Y | Y | Y | Y | Y | Y | N/A | Y |
| ^85^ | Y | N | Y | Y | Y | Y | Y | N | N/A | Y | Y | N/A |

| **Reference no.** | **12: Measure-ment and valuation of preference based outcomes** | **13a: Estimating resources and cost** | **13b: Estimating resources and cost** | **14: Currency, price date, and conversion** | **15: Choice of Model** | **16: Assumptions** | **17: Analytical methods** | **18: Study parameters** | **19: Incremental costs and outcomes** | **20a: Characteris-ing uncertainty** | **20b: Characteris-ing uncertainty** | **21: Characteris-ing heterogenity** |
| --- | --- | --- | --- | --- | --- | --- | --- | --- | --- | --- | --- | --- |
| ^2^ | N/A | N | N/A | N | N/A | N/A | N | Y | N | N | N/A | N/A |
| ^3^ | N/A | Y | N/A | N | N/A | N/A | N | Y | Y | N | N/A | N/A |
| ^4^ | Y | Y | N/A | Y | N/A | N/A | N | Y | Y | N | N/A | N/A |
| ^5^ | N/A | Y | N/A | N | N/A | N/A | N | Y | Y | N | N/A | N/A |
| ^6^ | Y | N/A | Y | Y | Y | Y | Y | Y | Y | N/A | Y | N/A |
| ^7^ | N/A | N/A | Y | Y | Y | Y | Y | Y | Y | N/A | Y | N/A |
| ^8^ | N/A | Y | N/A | N | N/A | N/A | N | Y | Y | N | N/A | N/A |
| ^9^ | N/A | N/A | Y | Y | Y | Y | Y | Y | Y | N/A | Y | N |
| ^10^ | Y | N/A | Y | Y | Y | Y | N | Y | Y | N/A | N | N/A |
| ^11^ | N/A | N/A | Y | Y | Y | Y | Y | Y | Y | N/A | Y | N/A |
| ^12^ | Y | N/A | Y | Y | Y | Y | Y | Y | Y | N/A | Y | N |
| ^13^ | N/A | N/A | Y | Y | N | Y | Y | Y | Y | N/A | Y | N |
| ^14^ | N/A | N/A | Y | Y | Y | Y | Y | Y | Y | N/A | Y | N/A |
| ^15^ | Y | N/A | Y | Y | Y | Y | Y | Y | Y | N/A | Y | N |
| ^16^ | Y | N/A | Y | Y | Y | Y | Y | Y | Y | N/A | Y | N |
| ^17^ | N/A | N/A | Y | Y | Y | Y | Y | Y | Y | N/A | Y | N |
| ^18^ | Y | N/A | Y | Y | Y | Y | Y | Y | Y | N/A | Y | N |
| ^19^ | N/A | N/A | Y | Y | Y | Y | Y | Y | Y | N/A | Y | N |
| ^20^ | N/A | N/A | Y | Y | Y | Y | Y | Y | Y | N/A | Y | Y |
| ^21^ | N/A | N | N/A | N | N/A | N/A | N | Y | Y | N | N/A | N/A |
| ^22^ | N/A | N/A | Y | Y | Y | Y | Y | Y | N | N/A | Y | N/A |
| ^23^ | N | N/A | Y | Y | Y | Y | Y | Y | Y | N/A | Y | Y |
| ^24^ | Y | N/A | Y | Y | Y | Y | Y | Y | Y | N/A | Y | N/A |
| ^25^ | Y | N/A | Y | Y | Y | Y | Y | Y | Y | N/A | Y | N/A |
| ^26^ | Y | N/A | Y | Y | Y | Y | N | Y | Y | N/A | Y | N |
| ^27^ | N/A | N/A | Y | N | Y | Y | N | Y | Y | N/A | Y | N/A |
| ^28^ | N/A | N/A | Y | Y | Y | Y | Y | Y | Y | N | Y | N/A |
| ^29^ | N/A | N | Y | Y | Y | Y | N | Y | Y | N/A | Y | N/A |
| ^30^ | Y | N/A | Y | Y | Y | Y | Y | Y | Y | N/A | Y | N/A |
| ^31^ | N/A | N/A | Y | Y | Y | Y | N | Y | Y | N/A | Y | N/A |
| ^32^ | N/A | N | N/A | Y | N/A | N/A | N | Y | Y | Y | N/A | N/A |
| ^33^ | Y | N/A | Y | Y | Y | Y | Y | Y | Y | N/A | Y | N |
| ^34^ | Y | N/A | Y | Y | Y | Y | Y | Y | Y | N/A | Y | N |
| ^35^ | N/A | N/A | Y | Y | Y | Y | Y | Y | Y | N/A | Y | N |
| ^36^ | N/A | N/A | Y | Y | Y | Y | Y | Y | Y | N/A | Y | N/A |
| ^37^ | Y | N/A | Y | Y | Y | Y | Y | Y | Y | N/A | Y | N |
| ^38^ | N/A | N/A | Y | N | Y | Y | N | N | N | N/A | Y | N |
| ^39^ | N/A | Y | N/A | Y | N/A | N/A | Y | Y | Y | N | N/A | N/A |
| ^40^ | N | N/A | N | Y | Y | Y | Y | Y | Y | N/A | Y | N/A |
| ^41^ | Y | N/A | N | N | Y | Y | Y | Y | Y | N/A | Y | N/A |
| ^42^ | N/A | N/A | Y | Y | N | Y | N | Y | Y | N/A | Y | N/A |
| ^43^ | N/A | N/A | Y | N | Y | Y | Y | Y | Y | N/A | Y | N |
| ^44^ | N/A | Y | N/A | Y | N/A | N/A | Y | Y | Y | Y | N/A | N/A |
| ^45^ | N/A | Y | N/A | Y | N/A | N/A | Y | Y | Y | Y | N/A | N/A |
| ^46^ | N/A | N/A | N | N | Y | N | Y | Y | N | N | N/A | N |
| ^47^ | N/A | Y | N/A | Y | N/A | N/A | Y | Y | Y | Y | N/A | N |
| ^48^ | N/A | N/A | Y | Y | N | Y | Y | Y | Y | N/A | Y | N/A |
| ^49^ | N/A | Y | N/A | Y | N/A | N/A | Y | Y | Y | Y | N/A | N/A |
| ^50^ | N/A | Y | N/A | Y | N/A | N/A | N | Y | Y | Y | N/A | N/A |
| ^51^ | N/A | N/A | Y | Y | N | Y | Y | Y | Y | N/A | N | N/A |
| ^52^ | N/A | Y | N/A | Y | N/A | N/A | N | Y | Y | Y | N/A | N/A |
| ^53^ | Y | Y | N/A | N | N/A | N/A | N | Y | Y | Y | N/A | N/A |
| ^54^ | Y | N/A | Y | N | Y | Y | N | Y | Y | N/A | Y | N/A |
| ^55^ | N/A | N | N/A | N | N/A | N/A | N | Y | Y | Y | N/A | N/A |
| ^56^ | N/A | N/A | Y | N | Y | Y | Y | Y | Y | N/A | Y | N/A |
| ^57^ | N | N/A | Y | Y | Y | Y | N | Y | Y | N/A | Y | N/A |
| ^58^ | N/A | Y | N/A | Y | N/A | N/A | N | Y | Y | N | N/A | N/A |
| ^59^ | N/A | N/A | Y | Y | Y | Y | Y | Y | Y | N/A | Y | Y |
| ^60^ | N/A | N | N/A | N | N/A | N/A | Y | Y | Y | N | N/A | N/A |
| ^61^ | Y | Y | N/A | N | N/A | N/A | Y | Y | Y | N | N/A | N/A |
| ^62^ | N/A | N/A | Y | Y | Y | Y | Y | Y | Y | N/A | Y | Y |
| ^63^ | Y | N | N/A | N | N/A | N/A | N | Y | Y | Y | N/A | N/A |
| ^64^ | N/A | Y | N/A | Y | N/A | N/A | Y | Y | N | N | N/A | N/A |
| ^65^ | N | Y | N/A | N | N/A | N/A | N | Y | Y | N | N/A | N/A |
| ^66^ | N | N | N/A | N | N/A | N/A | N | N | Y | N | N/A | N/A |
| ^67^ | N/A | N | N/A | Y | N/A | N/A | Y | Y | Y | N | N/A | N/A |
| ^68^ | Y | N/A | Y | Y | Y | Y | Y | Y | Y | N/A | Y | N |
| ^69^ | N/A | Y | N/A | N | N/A | N/A | N | Y | N | Y | N/A | N/A |
| ^70^ | N/A | N | N/A | N | N/A | N/A | N | Y | N | Y | N/A | N/A |
| ^71^ | N/A | Y | N/A | N | N/A | N/A | N | Y | Y | N | N/A | N/A |
| ^72^ | N/A | N | N/A | N | N/A | N/A | N | Y | N | Y | N/A | N/A |
| ^73^ | N/A | Y | N/A | N | N/A | N/A | N | Y | Y | Y | N/A | N/A |
| ^74^ | N/A | Y | N/A | Y | N/A | N/A | Y | Y | Y | Y | N/A | N/A |
| ^75^ | N/A | Y | N/A | N | N/A | N/A | N | Y | Y | N | N/A | N/A |
| ^76^ | N/A | N | N/A | N | N/A | N/A | N | N | Y | Y | N/A | N/A |
| ^77^ | N/A | Y | N/A | N | N/A | N/A | N | N | Y | Y | N/A | N/A |
| ^78^ | N/A | N | N/A | N | N/A | N/A | N | N | Y | N | N/A | N/A |
| ^79^ | N/A | N | N/A | N | N/A | N/A | Y | Y | N | Y | N/A | N/A |
| ^80^ | N/A | N | N/A | N | N/A | N/A | Y | Y | Y | Y | N/A | N/A |
| ^81^ | N/A | N | N/A | N | N/A | N/A | N | N | N | N | N/A | N/A |
| ^82^ | N/A | N | N/A | N | N/A | N/A | N | N | N | N | N/A | N/A |
| ^83^ | N/A | N | N/A | N | N/A | N/A | Y | Y | Y | N | N/A | N/A |
| ^84^ | N/A | N/A | Y | Y | N/A | N/A | Y | Y | Y | N/A | Y | N/A |
| ^85^ | N/A | Y | N/A | Y | N/A | N/A | Y | Y | Y | Y | N | N/A |

| **Reference no.** | **22: Study findings, limitations, generalizability, and current knowledge** | **23: Source of funding** | **24: CoI** | **Number of ‘Y’** | **Number of ‘N’** | **Composite quality score** |
| --- | --- | --- | --- | --- | --- | --- |
| ^2^ | Y | N | N | 10 | 9 | 0·53 |
| ^3^ | N | N | N | 10 | 10 | 0·50 |
| ^4^ | N | N | N | 12 | 7 | 0·63 |
| ^5^ | Y | N | N | 11 | 7 | 0·61 |
| ^6^ | Y | Y | Y | 23 | 0 | 1·00 |
| ^7^ | Y | Y | Y | 22 | 0 | 1·00 |
| ^8^ | N | Y | Y | 12 | 7 | 0·63 |
| ^9^ | Y | Y | Y | 21 | 2 | 0·91 |
| ^10^ | Y | Y | Y | 20 | 3 | 0·87 |
| ^11^ | Y | N | N | 19 | 3 | 0·86 |
| ^12^ | Y | Y | Y | 22 | 2 | 0·92 |
| ^13^ | Y | Y | Y | 20 | 3 | 0·87 |
| ^14^ | Y | Y | Y | 19 | 3 | 0·86 |
| ^15^ | Y | Y | Y | 21 | 3 | 0·88 |
| ^16^ | Y | Y | Y | 21 | 3 | 0·88 |
| ^17^ | Y | Y | Y | 21 | 2 | 0·91 |
| ^18^ | Y | Y | Y | 23 | 1 | 0·96 |
| ^19^ | Y | Y | Y | 20 | 3 | 0·87 |
| ^20^ | Y | Y | Y | 23 | 0 | 1·00 |
| ^21^ | N | Y | Y | 13 | 6 | 0·68 |
| ^22^ | Y | Y | Y | 19 | 3 | 0·86 |
| ^23^ | Y | Y | Y | 20 | 4 | 0·83 |
| ^24^ | Y | N | Y | 21 | 1 | 0·95 |
| ^25^ | Y | N | Y | 20 | 3 | 0·87 |
| ^26^ | Y | N | N | 20 | 4 | 0·83 |
| ^27^ | Y | Y | Y | 18 | 3 | 0·86 |
| ^28^ | Y | Y | Y | 19 | 4 | 0·83 |
| ^29^ | Y | Y | Y | 20 | 4 | 0·83 |
| ^30^ | Y | Y | N | 22 | 1 | 0·96 |
| ^31^ | Y | Y | Y | 20 | 2 | 0·91 |
| ^32^ | Y | Y | Y | 16 | 3 | 0·84 |
| ^33^ | Y | Y | Y | 23 | 1 | 0·96 |
| ^34^ | Y | Y | Y | 20 | 3 | 0·87 |
| ^35^ | Y | Y | Y | 21 | 2 | 0·91 |
| ^36^ | Y | Y | Y | 22 | 0 | 1·00 |
| ^37^ | Y | Y | Y | 20 | 4 | 0·83 |
| ^38^ | Y | Y | Y | 15 | 8 | 0·65 |
| ^39^ | Y | Y | Y | 16 | 3 | 0·84 |
| ^40^ | Y | Y | Y | 18 | 5 | 0·78 |
| ^41^ | Y | Y | Y | 19 | 4 | 0·83 |
| ^42^ | Y | N | N | 17 | 5 | 0·77 |
| ^43^ | Y | Y | N | 17 | 6 | 0·74 |
| ^44^ | Y | Y | Y | 19 | 1 | 0·95 |
| ^45^ | Y | N | N | 17 | 3 | 0·85 |
| ^46^ | Y | N | Y | 14 | 8 | 0·64 |
| ^47^ | Y | Y | Y | 18 | 3 | 0·86 |
| ^48^ | Y | Y | Y | 18 | 4 | 0·82 |
| ^49^ | Y | Y | N | 16 | 4 | 0·80 |
| ^50^ | Y | Y | Y | 16 | 3 | 0·84 |
| ^51^ | Y | Y | N | 16 | 5 | 0·76 |
| ^52^ | Y | Y | N | 15 | 4 | 0·79 |
| ^53^ | Y | Y | Y | 17 | 3 | 0·85 |
| ^54^ | Y | Y | Y | 19 | 4 | 0·83 |
| ^55^ | Y | Y | Y | 13 | 6 | 0·68 |
| ^56^ | Y | Y | Y | 20 | 1 | 0·95 |
| ^57^ | Y | Y | Y | 19 | 4 | 0·83 |
| ^58^ | Y | Y | Y | 15 | 5 | 0·75 |
| ^59^ | Y | Y | Y | 22 | 1 | 0·96 |
| ^60^ | N | Y | Y | 10 | 9 | 0·53 |
| ^61^ | N | N | Y | 14 | 6 | 0·70 |
| ^62^ | Y | Y | Y | 19 | 4 | 0·83 |
| ^63^ | Y | Y | Y | 14 | 6 | 0·70 |
| ^64^ | Y | Y | Y | 13 | 7 | 0·65 |
| ^65^ | N | Y | Y | 12 | 8 | 0·60 |
| ^66^ | N | Y | Y | 8 | 12 | 0·40 |
| ^67^ | N | Y | Y | 14 | 5 | 0·74 |
| ^68^ | Y | Y | N | 22 | 2 | 0·92 |
| ^69^ | N | N | Y | 11 | 8 | 0·58 |
| ^70^ | N | N | Y | 9 | 10 | 0·47 |
| ^71^ | Y | N | Y | 12 | 8 | 0·60 |
| ^72^ | N | Y | Y | 9 | 10 | 0·47 |
| ^73^ | N | N | Y | 11 | 8 | 0·58 |
| ^74^ | Y | Y | Y | 19 | 1 | 0·95 |
| ^75^ | Y | Y | N | 11 | 8 | 0·58 |
| ^76^ | N | N | N | 10 | 9 | 0·53 |
| ^77^ | Y | N | Y | 12 | 7 | 0·63 |
| ^78^ | N | N | N | 6 | 13 | 0·32 |
| ^79^ | N | N | N | 9 | 10 | 0·47 |
| ^80^ | Y | N | N | 11 | 8 | 0·58 |
| ^81^ | Y | N | N | 5 | 14 | 0·26 |
| ^82^ | Y | N | N | 4 | 15 | 0·21 |
| ^83^ | Y | N | Y | 12 | 7 | 0·63 |
| ^84^ | Y | Y | Y | 19 | 1 | 0·95 |
| ^85^ | Y | Y | N | 16 | 4 | 0·80 |

# **Source of evidence extraction result**

The source of evidence used for utilities, clinical effect sizes or adverse events and complications, baseline clinical data (if applicable), resource use, and costs in each study were extracted. The source of evidence was recorded according to Cooper et al. potential hierarchy of evidence (Table 6).^88^

Table 8. Source of evidence potential hierarchy according to Cooper et al., modified from Coyle and Lee ^88^

| **Rank** | **Data components** |
| --- | --- |
|  | *Clinical effect sizes, adverse events & complications* |
| 1+ | Meta-analysis of RCTs with direct comparison between comparator therapies, measuring final outcomes |
| 1 | Single RCT with direct comparison between comparator therapies, measuring final outcomes |
| 2+ | Meta-analysis of RCTs with direct comparison between comparator therapies, measuring surrogate outcomes  Meta-analysis of placebo-controlled RCTs with similar trial populations, measuring the final outcomes for each individual therapy |
| 2 | Single RCT with a direct comparison between comparator therapies, measuring surrogate outcomes  Single placebo-controlled RCTs with similar trial populations, measuring the final outcomes for each individual therapy |
| 3+ | Meta-analysis of placebo-controlled RCTs with similar trial populations, measuring the surrogate outcomes |
| 3 | Single placebo-controlled RCTs with similar trial populations, measuring the surrogate outcomes for each individual therapy |
| 4 | Observational studies (e.g., case control) |
| 5 | Non-analytic studies (e.g., case reports) |
| 6 | Expert opinion |
| 9 | Not stated |
|  | *Baseline clinical data* |
| 1 | Case series or analysis of reliable administrative databases specifically conducted for the study covering patients solely from the jurisdiction of interest |
| 2 | Recent case series or analysis of reliable administrative databases covering patients solely from the jurisdiction of interest |
| 3 | Recent case series or analysis of reliable administrative databases covering patients solely from another jurisdiction |
| 4 | Old case series or analysis of reliable administrative databases; estimate from RCTs |
| 5 | Estimates from previously published economic analyses: unsourced |
| 6 | Expert opinion |
| 9 | Not stated |
|  | *Resource use* |
| 1 | Prospective data collection or analysis of reliable administrative data for specific study |
| 2 | Recently published results of prospective data collection or recent analysis of reliable administrative data – same jurisdiction |
| 3 | Unsourced data from previous economic evaluations – same jurisdiction |
| 4 | Recently published results of prospective data collection or recent analysis of reliable administrative data – different jurisdiction |
| 5 | Unsourced data from previous economic evaluations – different jurisdiction |
| 6 | Expert opinion |
| 9 | Not stated |
|  | *Costs* |
| 1 | Cost calculations based on reliable databases or data sources conducted for specific study – same jurisdiction |
| 2 | Recently published cost calculations based on reliable databases or data course – same jurisdiction |
| 3 | Unsourced data from previous economic evaluation – same jurisdiction |
| 4 | Recently published cost calculations based on reliable databases or data course – different jurisdiction |
| 5 | Unsourced data from previous economic evaluation – different jurisdiction |
| 6 | Expert opinion |
| 9 | Not stated |
|  | *Utilities* |
| 1 | Direct utility assessment for the specific study from a sample either: (a) of the general population, (b) with knowledge of the disease(s) of interest, (c) of patients with the disease(s) of interest  Indirect utility assessment for the specific study from patient sample with disease(s) of interest, using a tool validated for the patient population |
| 2 | Indirect utility assessment for the specific study from patient sample with disease(s) of interest, using a tool not validated for the patient population |
| 3 | Direct utility assessment from a previous study from a sample either: (a) of the general population, (b) with knowledge of the disease(s) of interest, (c) of patients with the disease(s) of interest |
| 4 | Unsourced utility data from previous study – method of elicitation unknown |
| 5 | Patient preference values obtained from a visual analogue scale |
| 6 | Delphi panels, expert opinion |
| 9 | Not stated |

**Table 9. Source of evidence extraction result**

| **Reference no.** | **Utilities (if applicable)** | **Clinical effect sizes/adverse events and complications** | **Baseline clinical data (if applicable)** | **Resource use** | **Costs** |
| --- | --- | --- | --- | --- | --- |
| ^2^ | N/A | 4 | 9 | 9 | 9 |
| ^3^ | N/A | 4 | N/A | 1 | 1 |
| ^4^ | 2 | N/A | N/A | 1 | 1 |
| ^5^ | N/A | N/A | N/A | 1 | 1 |
| ^6^ | 9 | 5 | 2 | 2 | 2 |
| ^7^ | N/A | 5 | N/A | 2 | 2 |
| ^8^ | N/A | 4 | 9 | 1,2 | 1,2 |
| ^9^ | N/A | 2 | 2 | 2 | 2 |
| ^10^ | 3 | 1+,4 | 2 | 2 | 2 |
| ^11^ | N/A | 6 | 3,6 | 4 | 1,4 |
| ^12^ | 1 | 1+ | 2,3 | 9 | 2,4 |
| ^13^ | N/A | 5 | 9 | 1,2 | 1,2 |
| ^14^ | N/A | 9 | 9 | 2 | 4 |
| ^15^ | 3 | 5 | 9 | 2 | 1,2 |
| ^16^ | 1 | 2 | 4 | 1,2 | 1,2 |
| ^17^ | N/A | 9 | 2,3 | 2 | 2 |
| ^18^ | 3 | 4,5 | 3 | 1,4 | 1,4 |
| ^19^ | N/A | 1 | 2 | 2 | 2 |
| ^20^ | N/A | 1 | 2,3 | 1, 2 | 1,2 |
| ^21^ | N/A | 4 | 1 | 1 | 1 |
| ^22^ | N/A | 5 | 5 | 2 | 2 |
| ^23^ | 9 | 5 | 2 | 9 | 6 |
| ^24^ | 5 | 6 | 6 | 2 | 2 |
| ^25^ | 3 | 4,9 | N/A | 3 | 2 |
| ^26^ | 3 | 1+ | 2 | 2 | 2 |
| ^27^ | N/A | 5 | 2 | 6 | 2 |
| ^28^ | N/A | 6 | 6 | 2,6 | 2 |
| ^29^ | 9 | 1 | 9 | 4 | 2 |
| ^30^ | 3 | 4 | 2 | 9 | 2 |
| ^31^ | N/A | 5 | 1 | 4,6 | 2,6 |
| ^32^ | N/A | 4 | 2 | 9 | 9 |
| ^33^ | 3 | 2, 4,5 | 2,3 | 2 | 1,4 |
| ^34^ | 3 | N/A | 2 | 6 | 6 |
| ^35^ | N/A | 5 | 2 | 1 | 1 |
| ^36^ | N/A | 1+,5 | 4 | 1, 2 | 1,2 |
| ^37^ | 1 | 9 | 9 | 1 | 1 |
| ^38^ | N/A | 5 | 2 | 2 | 9 |
| ^39^ | N/A | 4 | 1 | 1 | 1 |
| ^40^ | 1 | 4 | 1 | 9 | 9 |
| ^41^ | 1 | 4 | 1 | 9 | 2 |
| ^42^ | N/A | 4 | N/A | 1 | 1 |
| ^43^ | N/A | 6 | 6 | 2 | 2 |
| ^44^ | N/A | 1 | N/A | 1 | 1,4 |
| ^45^ | N/A | 5 | 1 | 1 | 1 |
| ^46^ | N/A | 4 | 1 | 2 | 2 |
| ^47^ | N/A | 4 | 1 | 1 | 1 |
| ^48^ | N/A | 4 | 1 | 9 | 1 |
| ^49^ | N/A | 4 | 2 | 9 | 1 |
| ^50^ | N/A | 4 | 6 | 2 | 1 |
| ^51^ | N/A | 4 | 1 | 1,4 | 2,4 |
| ^52^ | N/A | 4 | 9 | 1 | 1 |
| ^53^ | 1 | 4 | 1 | 1 | 1 |
| ^54^ | 1 | 2+ | 1 | 2 | 2 |
| ^55^ | N/A | 4 | 2 | 9 | 1 |
| ^56^ | N/A | N/A | N/A | 2,6 | 4 |
| ^57^ | 3 | 4 | 4 | 2 | 2 |
| ^58^ | N/A | 4 | 2 | 2 | 2 |
| ^59^ | N/A | 2 | 2 | 1 | 1 |
| ^60^ | N/A | 4 | 1 | 2 | 1 |
| ^61^ | 1 | 4 | 1 | 9 | 2 |
| ^62^ | N/A | 4 | N/A | 5 | 4 |
| ^63^ | 1 | 4 | 1 | 9 | 9 |
| ^64^ | N/A | 4 | 1 | 1 | 1 |
| ^65^ | 1 | 4 | 9 | 1 | 1 |
| ^66^ | 9 | 4 | 9 | 9 | 2 |
| ^67^ | N/A | 4 | 9 | 9 | 9 |
| ^68^ | 3 | 1,2+ | 4 | 2 | 1 |
| ^69^ | N/A | 4 | 9 | 2 | 2 |
| ^70^ | N/A | 4 | 9 | 2 | 2 |
| ^71^ | N/A | 4 | 2 | 2 | 2 |
| ^72^ | N/A | 4 | 2 | 6 | 2 |
| ^73^ | N/A | 4 | 2 | 2 | 2 |
| ^74^ | N/A | 2 | N/A | 9 | 4 |
| ^75^ | N/A | 4 | 1 | 9 | 2 |
| ^76^ | N/A | 4 | 1 | 9 | 9 |
| ^77^ | N/A | 4 | 9 | 2 | 2 |
| ^78^ | N/A | 4 | 9 | 9 | 1 |
| ^79^ | N/A | 4 | 2 | 9 | 2 |
| ^80^ | N/A | 4 | 2 | 9 | 2 |
| ^81^ | N/A | 4 | 9 | 9 | 9 |
| ^82^ | N/A | 4 | 9 | 9 | 9 |
| ^83^ | N/A | 4 | 2 | 2 | 2 |
| ^84^ | N/A | 4 | N/A | 1 | 2 |
| ^85^ | N/A | 2 | 2 | 9 | 4 |

# **Regression analyses**

Fractional regression was chosen as the methodology and reporting composite quality scores containing observations equal to 1. Logit and probit models were applied for the univariate analyses to examine the association of possible predictors below. No difference in log pseudolikelihood result between logit and probit models for methodology and reporting scores as seen in Table 8. The logit model was chosen for the multivariable analyses.

A forward stepwise selection process was chosen because of the relatively small sample size. In each step, the possible predictors were included one by one from the highest to the lowest log pseudolikelihood from the univariate fractional logistic regression. To minimize the chance of omitting important predictors, variables were added until the newly added variable was not significant at 10%. For the methodology score, model 5 was chosen as the final model while model 3 was chosen as the final model for reporting score.

**Table 10. Log pseudolikelihood results from univariate fractional logistic and probit regression**

| Study characteristic | Methodology Score  Log Pseudolikelihood | | Reporting Score  Log Pseudolikelihood | |
| --- | --- | --- | --- | --- |
|  | **Logit** | **Probit** | **Logit** | **Probit** |
| Study design | -53·97 | -53·97 | -43·24 | -43·24 |
| Source of funding | -54·84 | -54·84 | -43·57 | -43·57 |
| Involvement of foreign affiliation | -54·07 | -54·07 | -43·90 | -43·90 |
| First author affiliation | -55·13 | -55·13 | -44·02 | -44·02 |
| Technology type | -55·55 | -55·55 | -45·29 | -45·29 |
| Disease | -55·72 | -55·72 | -46·07 | -46·07 |

**Table 11. Forward stepwise regression models for methodology score**

| Study characteristic |  | P value | | | | | |
| --- | --- | --- | --- | --- | --- | --- | --- |
|  |  | Model 1 | Model 2 | Model 3 | Model 4 | Model 5 | Model 6 |
| Study design | Clinical study based | Ref | Ref | Ref | Ref | Ref | Ref |
|  | Model based | <0·00 | 0·00 | <0·00 | <0·00 | <0·00 | 0·00 |
| Involvement of foreign affiliation | No |  | Ref | Ref | Ref | Ref | Ref |
|  | Yes |  | 0·00 | 0·02 | 0·00 | 0·00 | 0·01 |
| Source of funding | No funding or not stated |  |  | Ref |  |  |  |
|  | Government |  |  | 0·77 |  |  |  |
|  | Private |  |  | 0·53 |  |  |  |
|  | Mix (government and private) |  |  | 0·10 |  |  |  |
| First author affiliation | Foreign or mix |  |  |  | Ref |  |  |
|  | Indonesian |  |  |  | 0·18 |  |  |
| Technology type | Drug |  |  |  |  | Ref |  |
|  | Biological matter |  |  |  |  | 0·53 |  |
|  | Support system |  |  |  |  | 0·33 |  |
|  | Others |  |  |  |  | 0·06 |  |
| Disease | Top 10 burdens by DALY |  |  |  |  |  | Ref |
|  | Non-top 10 burdens by DALY |  |  |  |  |  | 0·72 |

**Table 12. Forward stepwise regression models for reporting score**

| Study characteristic |  | P value | | | | | |
| --- | --- | --- | --- | --- | --- | --- | --- |
|  |  | Model 1 | Model 2 | Model 3 | Model 4 | Model 5 | Model 6 |
| Study design | Clinical study based | Ref | Ref | Ref | Ref | Ref | Ref |
|  | Model based | <0·00 | <0·00 | <0·00 | <0·00 | <0·00 | <0·00 |
| Source of funding | No funding or not stated |  | Ref | Ref | Ref | Ref | Ref |
|  | Government |  | 0·01 | 0·01 | 0·01 | 0·01 | 0·01 |
|  | Private |  | 0·01 | 0·02 | 0·03 | 0·02 | 0·02 |
|  | Mix (government and private) |  | <0·00 | 0·00 | <0·00 | 0·00 | 0·00 |
| Involvement of foreign affiliation | No |  |  | Ref | Ref | Ref | Ref |
|  | Yes |  |  | 0·06 | 0·45 | 0·06 | 0·06 |
| First author affiliation | Foreign or mix |  |  |  | Ref |  |  |
|  | Indonesian |  |  |  | 0·14 |  |  |
| Technology type | Drug |  |  |  |  | Ref |  |
|  | Biological matter |  |  |  |  | 0·64 |  |
|  | Support system |  |  |  |  | 0·99 |  |
|  | Others |  |  |  |  | 0·53 |  |
| Disease | Top 10 burdens by DALY |  |  |  |  |  | Ref |
|  | Non-top 10 burdens by DALY |  |  |  |  |  | 0·98 |

**References**

1. Indonesia Health Technology Assessment Committee (InaHTAC). Health Technology Assessment (HTA) Guideline. Jakarta: Ministry of Health; 2017. <http://adphealth.org>. Accessed 9 September 2021.

2. Rahmawati C, Nurwahyuni A. Cost minimization analysis for anti-hypertensive drug between the combination of Ramipiril-Spironolactone and Valsartan of congestive heart failure patients in XY public hospital, Jakarta, 2014. *Jurnal Ekonomi Kesehatan Indonesia*. 2017; **1**.

3. Rahmiyati AL, Abdillah AD, Susilowati., Anggraini D. Cost benefit analysis program pemberian makanan tambahan (PMT) susu pada karyawan di PT. Trisula Textile Industries Tbk. Cimahi tahun 2018. *Jurnal Ekonomi Kesehatan Indonesia*. 2018; **3**.

4. Novelia E, Nugraha RR, Thabrany H. Cost effectiveness analysis between hemodialysis. *Jurnal Ekonomi Kesehatan Indonesia*. 2017; **1**.

5. Merliana H, Sjaaf AC. Cost minimization analysis of generic and branded Amlodipin. *Jurnal Ekonomi Kesehatan Indonesia*. 2017; **1(3)**.

6. Machlaurin A, Dolk FCK, Setiawan D, van der Werf TS, Postma MJ. Cost-Effectiveness Analysis of BCG Vaccination against Tuberculosis in Indonesia: A Model-Based Study. *Vaccines (Basel)*. 2020; **8(4)**. <https://www.ncbi.nlm.nih.gov/pubmed/33256143>.

7. Zakiyah N, van Asselt ADI, Setiawan D, Cao Q, Roijmans F, Postma MJ. Cost-Effectiveness of Scaling Up Modern Family Planning Interventions in Low- and Middle-Income Countries: An Economic Modeling Analysis in Indonesia and Uganda. *Appl Health Econ Health Policy*. 2019; **17(1)**: 65-76. <https://www.ncbi.nlm.nih.gov/pubmed/30178267>.

8. Rochmah TN, Wulandari A, Dahlui M, Ernawaty, Wulandari RD. Cost Effectiveness Analysis Using Disability-Adjusted Life Years for Cataract Surgery. *Int J Environ Res Public Health*. 2020; **17(16)**. <https://www.ncbi.nlm.nih.gov/pubmed/32824872>.

9. Brady OJ, Kharisma DD, Wilastonegoro NN, et al. The cost-effectiveness of controlling dengue in Indonesia using wMel Wolbachia released at scale: a modelling study. *BMC Med*. 2020; **18(1)**: 186. <https://www.ncbi.nlm.nih.gov/pubmed/32641039>.

10. Suwantika AA, Kautsar AP, Supadmi W, et al. Cost-Effectiveness of Dengue Vaccination in Indonesia: Considering Integrated Programs with Wolbachia-Infected Mosquitos and Health Education. *Int J Environ Res Public Health*. 2020; **17(12)**. <https://www.ncbi.nlm.nih.gov/pubmed/32545688>.

11. Broughton EI. Economic evaluation of Haemophilus influenzae type B vaccination in Indonesia: a cost-effectiveness analysis. *J Public Health (Oxf)*. 2007; **29(4)**: 441-8. <https://www.ncbi.nlm.nih.gov/pubmed/17875589>.

12. Suwantika AA, Supadmi W, Ali M, Abdulah R. Cost-effectiveness and budget impact analyses of dengue vaccination in Indonesia. *PLoS Negl Trop Dis*. 2021; **15(8)**: e0009664. <https://www.ncbi.nlm.nih.gov/pubmed/34383764>.

13. McPake B, Edoka I, Witter S, et al. Cost-effectiveness of community-based practitioner programmes in Ethiopia, Indonesia and Kenya. *Bull World Health Organ*. 2015; **93(9)**: 631-9A. <https://www.ncbi.nlm.nih.gov/pubmed/26478627>.

14. Emmett SD, Sudoko CK, Tucci DL, et al. Expanding Access: Cost-effectiveness of Cochlear Implantation and Deaf Education in Asia. *Otolaryngol Head Neck Surg*. 2019; **161(4)**: 672-82. <https://www.ncbi.nlm.nih.gov/pubmed/31210566>.

15. Setiawan D, Andrijono, Hadinegoro SR, et al. Cervical cancer prevention in Indonesia: An updated clinical impact, cost-effectiveness and budget impact analysis. *PLoS One*. 2020; **15(3)**: e0230359. <https://www.ncbi.nlm.nih.gov/pubmed/32203527>.

16. Lilyasari O, Subekti Y, Atika N, et al. Economic evaluation of sildenafil for the treatment of pulmonary arterial hypertension in Indonesia. *BMC Health Serv Res*. 2019; **19(1)**: 573. <https://www.ncbi.nlm.nih.gov/pubmed/31412857>.

17. Trickey A, Hiebert L, Perfect C, et al. Hepatitis C virus elimination in Indonesia: Epidemiological, cost and cost-effectiveness modelling to advance advocacy and strategic planning. *Liver Int*. 2020; **40(2)**: 286-97. <https://www.ncbi.nlm.nih.gov/pubmed/31454466>.

18. Suwantika AA, Zakiyah N, Abdulah R, et al. Cost-Effectiveness and Budget Impact Analyses of Pneumococcal Vaccination in Indonesia. *J Environ Public Health*. 2021; **2021**: 7494965. <https://www.ncbi.nlm.nih.gov/pubmed/33995536>.

19. Devine A, Howes RE, Price DJ, et al. Cost-Effectiveness Analysis of Sex-Stratified Plasmodium vivax Treatment Strategies Using Available G6PD Diagnostics to Accelerate Access to Radical Cure. *Am J Trop Med Hyg*. 2020; **103(1)**: 394-403. <https://www.ncbi.nlm.nih.gov/pubmed/32372747>.

20. Paintain L, Hill J, Ahmed R, et al. Cost-effectiveness of intermittent preventive treatment with dihydroartemisinin-piperaquine versus single screening and treatment for the control of malaria in pregnancy in Papua, Indonesia: a provider perspective analysis from a cluster-randomised trial. *The Lancet Global Health*. 2020; **8(12)**: e1524-e33. <https://doi.org/10.1016/s2214-109x(20)30386-7>.

21. Baroroh F, Sari A, Masruroh N. Cost Effectiveness Analysis of Candesartan Therapy in Comparison to Candesartan-Amlodipine Therapy on Hypertensive Outpatients. *Open Access Maced J Med Sci*. 2019; **7(22)**: 3837-40. <https://www.ncbi.nlm.nih.gov/pubmed/32127987>.

22. Tromp N, Siregar A, Barnabas L, et al. Cost-effectiveness of scaling up voluntary counselling and testing in West-Java, Indonesia. *Acta Medica Indonesiana - The Indonesian Journal of Internal Medicine*. 2013; **45**.

23. Kosen S, Andrijono A, Ocviyanti D, Indriatmi W. The Cost-Effectiveness of Quadrivalent Human Papillomavirus Vaccination in Indonesia. *Asian Pac J Cancer Prev*. 2017; **18(7)**: 2011-7. <https://www.ncbi.nlm.nih.gov/pubmed/28749644>.

24. Chanthawong S, Lim YH, Subongkot S, et al. Cost-effectiveness analysis of olanzapine-containing antiemetic therapy for managing highly emetogenic chemotherapy in Southeast Asia: a multinational study. *Support Care Cancer*. 2019; **27(3)**: 1109-19. <https://www.ncbi.nlm.nih.gov/pubmed/30112718>.

25. Suwantika AA, Beutels P, Postma MJ. Cost-effectiveness of hepatitis A vaccination in Indonesia. *Hum Vaccin Immunother*. 2014; **10(8)**: 2342-9. <https://www.ncbi.nlm.nih.gov/pubmed/25424941>.

26. Suwantika AA, Tu HA, Postma MJ. Cost-effectiveness of rotavirus immunization in Indonesia: taking breastfeeding patterns into account. *Vaccine*. 2013; **31(32)**: 3300-7. <https://www.ncbi.nlm.nih.gov/pubmed/23707163>.

27. Suwantika AA, Zakiyah N, Puspitasari IM, Abdulah R. Cost-Effectiveness of Contraceptive Use in Indonesia after the Implementation of the National Health Insurance System. *J Pregnancy*. 2021; **2021**: 3453291. <https://www.ncbi.nlm.nih.gov/pubmed/34046230>.

28. Wera E, Mourits MCM, Siko MM, Hogeveen H. Cost-Effectiveness of Mass Dog Vaccination Campaigns against Rabies in Flores Island, Indonesia. *Transbound Emerg Dis*. 2017; **64(6)**: 1918-28. <https://www.ncbi.nlm.nih.gov/pubmed/27878980>.

29. Setiawan D, Dolk FC, Suwantika AA, Westra TA, JC WI, Postma MJ. Cost-Utility Analysis of Human Papillomavirus Vaccination and Cervical Screening on Cervical Cancer Patient in Indonesia. *Value Health Reg Issues*. 2016; **9**: 84-92. <https://www.ncbi.nlm.nih.gov/pubmed/27881267>.

30. Zakiyah N, Sinuraya RK, Kusuma ASW, Suwantika AA, Lestari K. Cost-Effectiveness Analysis of Sacubitril/Valsartan Compared to Enalapril for Heart Failure Patients in Indonesia. *Clinicoecon Outcomes Res*. 2021; **13**: 863-72. <https://www.ncbi.nlm.nih.gov/pubmed/34675566>.

31. Jeuland M, Cook J, Poulos C, Clemens J, Whittington D, Group DCES. Cost-effectiveness of new-generation oral cholera vaccines: a multisite analysis. *Value Health*. 2009; **12(6)**: 899-908. <https://www.ncbi.nlm.nih.gov/pubmed/19824189>.

32. Puspitasari IM, Legianawati D, Sinuraya RK, Suwantika AA. Cost-Effectiveness Analysis of Chemoradiation and Radiotherapy Treatment for Stage IIB and IIIB Cervical Cancer Patients. *Int J Womens Health*. 2021; **13**: 221-9. <https://www.ncbi.nlm.nih.gov/pubmed/33642882>.

33. Suwantika AA, Postma M. Effect of breastfeeding promotion interventions on cost-effectiveness of rotavirus immunization in Indonesia. *BMC Public Health*. 2013; **13**.

34. Home P, Baik SH, Galvez GG, Malek R, Nikolajsen A. An analysis of the cost-effectiveness of starting insulin detemir in insulin-naive people with type 2 diabetes. *J Med Econ*. 2015; **18(3)**: 230-40. <https://www.ncbi.nlm.nih.gov/pubmed/25407031>.

35. Wera E, Mourits MCM, Hogeveen H. Cost-effectiveness of mass dog rabies vaccination strategies to reduce human health burden in Flores Island, Indonesia. *Vaccine*. 2017; **35(48 Pt B)**: 6727-36. <https://www.ncbi.nlm.nih.gov/pubmed/29079100>.

36. Angell B, Lung T, Praveen D, et al. Cost-effectiveness of a mobile technology-enabled primary care intervention for cardiovascular disease risk management in rural Indonesia. *Health Policy Plan*. 2021; **36(4)**: 435-43. <https://www.ncbi.nlm.nih.gov/pubmed/33712844>.

37. Afiatin, Khoe LC, Kristin E, et al. Economic evaluation of policy options for dialysis in end-stage renal disease patients under the universal health coverage in Indonesia. *PLoS One*. 2017; **12(5)**: e0177436. <https://www.ncbi.nlm.nih.gov/pubmed/28545094>.

38. Joesoef MR, Remington PL, Tjiptoherijanto P. Epidemiological model and cost-effectiveness analysis of tuberculosis treatment programmes in Indonesia. *International Journal of Epidemiology*. 1989; **18**.

39. Endaryanto A, Nugraha RA. Indonesia-Based Study of the Clinical and Cost-Saving Benefits of Subcutaneous Allergen Immunotherapy for Children with Allergic Rhinitis in Private Practice. *Cells*. 2021; **10(7)**. <https://www.ncbi.nlm.nih.gov/pubmed/34360010>.

40. Gupta V, Baabbad R, Hammerby E, Nikolajsen A, Shafie AA. An analysis of the cost-effectiveness of switching from biphasic human insulin 30, insulin glargine, or neutral protamine Hagedorn to biphasic insulin aspart 30 in people with type 2 diabetes. *J Med Econ*. 2015; **18(4)**: 263-72. <https://www.ncbi.nlm.nih.gov/pubmed/25426701>.

41. Shafie AA, Gupta V, Baabbad R, Hammerby E, Home P. An analysis of the short- and long-term cost-effectiveness of starting biphasic insulin aspart 30 in insulin-naive people with poorly controlled type 2 diabetes. *Diabetes Res Clin Pract*. 2014; **106(2)**: 319-27. <https://www.ncbi.nlm.nih.gov/pubmed/25305133>.

42. Liu W, Clemens JD, Kari K, Xu ZY. Cost-effectiveness of Japanese encephalitis (JE) immunization in Bali, Indonesia. *Vaccine*. 2008; **26(35)**: 4456-60. <https://www.ncbi.nlm.nih.gov/pubmed/18602436>.

43. Wammes JJ, Siregar AY, Hidayat T, et al. Cost-effectiveness of methadone maintenance therapy as HIV prevention in an Indonesian high-prevalence setting: a mathematical modeling study. *Int J Drug Policy*. 2012; **23(5)**: 358-64. <https://www.ncbi.nlm.nih.gov/pubmed/22884538>.

44. Gessner BD, Sedyaningsih ER, Griffiths UK, et al. Vaccine-preventable haemophilus influenza type B disease burden and cost-effectiveness of infant vaccination in Indonesia. *Pediatr Infect Dis J*. 2008; **27(5)**: 438-43. <https://www.ncbi.nlm.nih.gov/pubmed/18398383>.

45. Cook J, Jeuland M, Whittington D, et al. The cost-effectiveness of typhoid Vi vaccination programs: calculations for four urban sites in four Asian countries. *Vaccine*. 2008; **26(50)**: 6305-16. <https://www.ncbi.nlm.nih.gov/pubmed/18835415>.

46. Farani M, Saldi SRF, Maulahela H, Abdullah M, Syam AF, Makmum D. Survival, stent patency, and cost-effectiveness of plastic biliary stent versus metal biliary stent for palliation in malignant biliary obstruction in a developing country tertiary hospital. *JGH Open*. 2021; **5(8)**: 959-65. <https://www.ncbi.nlm.nih.gov/pubmed/34386606>.

47. Zelman BW, Baral R, Zarlinda I, et al. Costs and cost-effectiveness of malaria reactive case detection using loop-mediated isothermal amplification compared to microscopy in the low transmission setting of Aceh Province, Indonesia. *Malar J*. 2018; **17(1)**: 220. <https://www.ncbi.nlm.nih.gov/pubmed/29859081>.

48. Johns B, Probandari A, Mahendradhata Y, Ahmad RA. An analysis of the costs and treatment success of collaborative arrangements among public and private providers for tuberculosis control in Indonesia. *Health Policy*. 2009; **93(2-3)**: 214-24. <https://www.ncbi.nlm.nih.gov/pubmed/19729220>.

49. Mahendradhata Y, Probandari A, Ahmad RA, et al. The incremental cost-effectiveness of engaging private practitioners to refer tuberculosis suspects to DOTS services in Jogjakarta, Indonesia. *Am J Trop Med Hyg*. 2010; **82(6)**: 1131—9. <https://doi.org/10.4269/ajtmh.2010.09-0447>.

50. Walker D, McDermott J, Fox-Rushby J, et al. An economic analysis of midwifery training programmes in South Kalimantan, Indonesia. *Bulletin of the World Health Organization*. 2002; **2002(1)**.

51. Friedman HB, Gift TL, Susanti I, Wasserheit JN. Risk-based versus alternative algorithms for antibiotic prophylaxis among women seeking early suction abortion: a cost-effectiveness simulation. *Sexually Transmitted Diseases*. 2001; **28(12)**: 714-24.

52. Berman P, Quinley J, Burhannuddin Y, et al. Maternal tetanus immunization in Aceh Province, Sumatra: the cost-effectiveness of alternative strategies. *Soc Sci Med*. 1991; **33**: 185-92.

53. Rakanita Y, Syamsunarno M, Sinuraya RK, Suradji EW, Abdulah R, Suwantika AA. Cost-Effectiveness of Ferrous Fumarate-Folic Acid and Ferrous Gluconate-Multivitamins in a High Prevalence Area of Iron Deficiency Anemia in Indonesia. *Ther Clin Risk Manag*. 2021; **17**: 1075-81. <https://www.ncbi.nlm.nih.gov/pubmed/34629872>.

54. Kristin E, Endarti D, Khoe LC, et al. Economic Evaluation of Adding Bevacizumab to Chemotherapy for Metastatic Colorectal Cancer (mCRC) Patients in Indonesia. *Asian Pac J Cancer Prev*. 2021; **22(6)**: 1921—26. <https://doi.org/10.31557/APJCP.2021.22.6.1921>.

55. Nasution A, Syed Sulaiman SA, Shafie AA. Cost-Effectiveness of Clinical Pharmacy Education on Infection Management among Patients with Chronic Kidney Disease in an Indonesian Hospital. *Value Health Reg Issues*. 2013; **2(1)**: 43-7. <https://www.ncbi.nlm.nih.gov/pubmed/29702851>.

56. Suwantika AA, Kautsar AP, Zakiyah N, Abdulah R, Boersma C, Postma MJ. Cost-Effectiveness Analysis of Spending on Research and Development to Address the Needs for Innovative Therapeutic Products in Indonesia. *Ther Clin Risk Manag*. 2020; **16**: 969-77. <https://www.ncbi.nlm.nih.gov/pubmed/33116546>.

57. Yuliwulandari R, Shin JG, Kristin E, et al. Cost-effectiveness analysis of genotyping for HLA-B*15:02 in Indonesian patients with epilepsy using a generic model. *Pharmacogenomics J*. 2021; **21(4)**: 476-83. <https://www.ncbi.nlm.nih.gov/pubmed/33824430>.

58. Priyadi A, Permana H, Muhtadi A, Sumiwi SA, Sinuraya RK, Suwantika AA. Cost-Effectiveness Analysis of Type 2 Diabetes Mellitus (T2DM) Treatment in Patients with Complications of Kidney and Peripheral Vascular Diseases in Indonesia. *Healthcare*. 2021; **9(2)**: 1—9. <https://doi.org/10.3390/healthcare9020211>.

59. Purba AKR, Ascobat P, Muchtar A, et al. Cost-Effectiveness Of Culture-Based Versus Empirical Antibiotic Treatment For Hospitalized Adults With Community-Acquired Pneumonia In Indonesia: A Real-World Patient-Database Study. *Clinicoecon Outcomes Res*. 2019; **11**: 729-39. <https://www.ncbi.nlm.nih.gov/pubmed/31819563>.

60. Lorensia A, Jalmav MMA. Cost-effectiveness analysis on the treatment of dengue fever in inpatients. *Teikyo Medical Journal*. 2021; **44(5)**.

61. Karaeng ND, Makhmud AI, Liaury K. The use of risperidone-combination and haloperidol-combination in schizophrenia patients: A cost utility analysis in psychiatric hospital of Prof. V.L. Ratumbuysang. *Medicina Clínica Práctica*. 2021; **4**. <https://www.sciencedirect.com/science/article/pii/S2603924921000471?via%3Dihub>.

62. Jo Y, Gomes I, Flack J, et al. Cost-effectiveness of scaling up short course preventive therapy for tuberculosis among children across 12 countries. *EClinicalMedicine*. 2021; **31**: 100707. <https://www.ncbi.nlm.nih.gov/pubmed/33554088>.

63. Sari SP, Funna ED, Septini R. Cost-utility analysis of tuberculosis patients with directly observed treatment and self-administered treatment in an army hospital, Indonesia. *Pharmaceutical Sciences Asia*. 2020; **47(3)**: 253-61.

64. Sinuraya RK, Aini AN, Rahayu C, Wathoni N, Abdulah R. The Effectiveness of Postoperative Antibiotics following Appendectomy in Pediatric Patients: A Cost Minimization Analysis. *The Open Public Health Journal*. 2020; **13(1)**: 80-6. <https://openpublichealthjournal.com/contents/volumes/V13/TOPHJ-13-80/TOPHJ-13-80.pdf>.

65. Wulandari A, Dahlui M, Ernawaty., Wulandari RD, Rochmah TN. Cost effectiveness analysis between small incision cataract surgery and phacoemulsification. *Journal of University of Malaya Medical Centre*. 2020; **23**.

66. Riwu M, Yubiliana G, Halimah E, Diantini A. Cost-Effectiveness Analysis of Pharmacist Counselling in Therapeutic Success and Quality of Life of Hypertensive Patients. *Research Journal of Pharmacy and Technology*. 2019; **12(12)**. <https://www.indianjournals.com/ijor.aspx?target=ijor:rjpt&volume=12&issue=12&article=036>.

67. Ulya F, Thabrany H, Nadjib M, Junadi P. Cost effectiveness of interventions using the TB DOTS strategy in public healthcare and private hospitals in Depok, West Java, Indonesia. *Indian Journal of Public Health Research & Development*. 2019; **10(9)**.

68. Dwiprahasto I, Kristin E, Endarti D, et al. Cost Effectiveness Analysis of Rivaroxaban Compared to Warfarin and Aspirin for Stroke Prevention Atrial Fibrillation (SPAF) in the Indonesian healthcare setting. *Indonesian Journal of Pharmacy*. 2019; **30(1)**. <https://indonesianjpharm.farmasi.ugm.ac.id/index.php/3/article/download/1507/896>.

69. Sari SP, Putri IDA, Nursanti B. Cost-Effectiveness Analysis of Ceftriaxone Generic and Patent in Children with Typhoid. *International Journal of Applied Pharmaceutics*. 2018; **10(1)**. <https://innovareacademics.in/journals/index.php/ijap/article/download/31426/16511>.

70. Purbandini CS, Sauriasari R. Cost-Effectiveness Analysis of Ceftriaxone and Non-Ceftriaxone on Typhoid Fever Patients. *International Journal of Applied Pharmaceutics*. 2018; **10(1)**. <https://innovareacademics.in/journals/index.php/ijap/article/download/31428/16513>.

71. Nafrialdi N, Handini NM, Instiaty I, Wijaya IP. A cost-effectiveness and safety analysis of dual antiplatelet therapy comparing aspirin–clopidogrel to aspirin–ticagrelor in patients with acute coronary syndrome. *Medical Journal of Indonesia*. 2018; **27(4)**: 262-70. <https://mji.ui.ac.id/journal/index.php/mji/article/download/3024/1264>.

72. Sanif R, Sulistiadi W, Manan H, Hidayat R. Cost-effectiveness analysis on advanced cervical carcinoma patients at Dr Mohammad Hoesin general hospital Palembang, South Sumatra, Indonesia. *Journal of South Asian Federation of Obstetrics and Gynaecology*. 2019; **10**: 316-20.

73. Chairunnisa HG, Sauriasari R, Rizkyani NA. Cost-effectiveness Analysis of Deferiprone and Deferasirox on Thalassemia Major Patients in Tangerang District Hospital, Indonesia. *Journal of Young Pharmacists*. 2018; **10(2s)**: S128-S31. <https://www.jyoungpharm.org/sites/default/files/JYoungPharm_10_2_s128.pdf>.

74. Zeng W, Halasa-Rappel YA, Baurin N, Coudeville L, Shepard DS. Cost-effectiveness of dengue vaccination in ten endemic countries. *Vaccine*. 2018; **36(3)**: 413-20. <https://www.ncbi.nlm.nih.gov/pubmed/29229427>.

75. Larasanty LPF, Sarasmita MA, Putra IGNAD. Cost-Effectiveness Analysis of Insulin Regimen on Type 2 Diabetes Mellitus Outpatient in Denpasar Municipality. *Asian Journal of Pharmaceutical and Clinical Research*. 2018; **11(1)**. <https://innovareacademics.in/journals/index.php/ajpcr/article/download/18695/13626>.

76. Sihombing YR, Nasution A, Rosidah R. Economic Impact of Counseling on the Management of Patients with Type 2 Diabetes Mellitus Admitted to a Hospital. *Asian Journal of Pharmaceutical and Clinical Research*. 2018; **11(13)**. <https://innovareacademics.in/journals/index.php/ajpcr/article/download/26577/14449>.

77. Ramadhani D, Harahap U, Nasution A. Cost-Effectiveness Analysis of Counseling in Therapy for Outpatients with Hypertension. *Asian Journal of Pharmaceutical and Clinical Research*. 2018; **11(13)**. <https://innovareacademics.in/journals/index.php/ajpcr/article/download/26555/14496>.

78. Laelasari E, Sauriasari R, Banun A. Cost-Effectiveness Analysis of Insulin, Sulfonylurea, and Sulfonylurea– Metformin in Type 2 Diabetes Mellitus. *Asian Journal of Pharmaceutical and Clinical Research*. 2017; **10(17)**. <https://innovareacademics.in/journals/index.php/ajpcr/article/download/23094/12894>.

79. Suratini S, Sauriasari R, Hamadah F. Cost-Effectiveness Analysis of Ceftriaxone-Azithromycin Combination and Single Levofloxacin as Empirical Antibiotics in Community-Acquired Pneumonia Inpatients at Persahabatan Hospital. *Asian Journal of Pharmaceutical and Clinical Research*. 2017; **10(17)**. <https://innovareacademics.in/journals/index.php/ajpcr/article/download/23112/12908>.

80. Ningsih F, Sauriasari R, Saptaningsih AB. Cost-Effectiveness Analysis on the Use of Parenteral Nutrition with D10-Ca Gluconate and D5 1/4ns in Normal-Weight Neonates with Respiratory Distress Syndrome. *International Journal of Applied Pharmaceutics*. 2017; **9**. <https://innovareacademics.in/journals/index.php/ijap/article/download/23297/12848>.

81. Destiani DP, Milanda T, Susilawati S, et al. Cost-Effectiveness Analysis of Ceftazidime-Levofloxacin and Cefotaxime-Erythromycin as Empirical Antibiotic Combinations in Respiratory Infection-Induced Sepsis. *Asian Journal of Pharmaceutical and Clinical Research*. 2017; **10(14)**. <https://innovareacademics.in/journals/index.php/ajpcr/article/download/19515/11408>.

82. Sinuraya RK, Gondodiputro S, Djuhaeni H. Proton Pump Inhibitors for Stress Ulcer Bleeding Prophylaxis in Critically Ill Patients: A Cost Analysis Study. *Asian Journal of Pharmaceutical and Clinical Research*. 2017; **10(14)**. <https://innovareacademics.in/journals/index.php/ajpcr/article/download/20413/12157>.

83. Pribadi FW, Dwiprahasto I, Thobari JA. Cost analysis of combination diuretic therapy with ace-inhibitors to diuretic therapy without ace-inhibitors in heart failure patients. *Bangladesh Journal of Medical Science*. 2016; **15(3)**: 368-75.

84. Wilopo SA, Kilgore P, Kosen S, et al. Economic evaluation of a routine rotavirus vaccination programme in Indonesia. *Vaccine*. 2009; **27 Suppl 5**: F67-74. <https://www.ncbi.nlm.nih.gov/pubmed/19931723>.

85. Lubell Y, Yeung S, Dondorp AM, et al. Cost-effectiveness of artesunate for the treatment of severe malaria. *Trop Med Int Health*. 2009; **14(3)**: 332-7. <https://www.ncbi.nlm.nih.gov/pubmed/19187518>.

86. GDP Compare Data Visualization. IHME, University of Washington. 2022. <https://vizhub.healthdata.org/gbd-compare/>. Accessed 17 May 2022.

87. Husereau D, Drummond M, Petrou S, et al. Consolidated Health Economic Evaluation Reporting Standards (CHEERS) statement. *Int J Technol Assess Health Care*. 2013; **29(2)**: 117—22. <https://doi.org/10.1017/S0266462313000160>.

88. Cooper N, Coyle D, Abrams K, Mugford M, Sutton A. Use of Evidence in Decision Models: An Appraisal of Health Technology Assessment in the UK since 1997. *J Health Serv Res Policy*. 2005; **10(4)**: 245—50. <http://doi.org/10.1258/135581905774414187>.
